# The Equivalence of Information-Theoretic and Likelihood-Based Methods for Neural Dimensionality Reduction

**DOI:** 10.1371/journal.pcbi.1004141

**Published:** 2015-04-01

**Authors:** Ross S. Williamson, Maneesh Sahani, Jonathan W. Pillow

**Affiliations:** 1 Gatsby Computational Neuroscience Unit, University College London, London, UK; 2 Centre for Mathematics and Physics in the Life Sciences and Experimental Biology, University College London, London, UK; 3 Princeton Neuroscience Institute, Department of Psychology, Princeton University, Princeton, New Jersey, USA; University of Tübingen and Max Planck Institute for Biologial Cybernetics, GERMANY

## Abstract

Stimulus dimensionality-reduction methods in neuroscience seek to identify a low-dimensional space of stimulus features that affect a neuron’s probability of spiking. One popular method, known as maximally informative dimensions (MID), uses an information-theoretic quantity known as “single-spike information” to identify this space. Here we examine MID from a model-based perspective. We show that MID is a maximum-likelihood estimator for the parameters of a linear-nonlinear-Poisson (LNP) model, and that the empirical single-spike information corresponds to the normalized log-likelihood under a Poisson model. This equivalence implies that MID does not necessarily find maximally informative stimulus dimensions when spiking is not well described as Poisson. We provide several examples to illustrate this shortcoming, and derive a lower bound on the information lost when spiking is Bernoulli in discrete time bins. To overcome this limitation, we introduce model-based dimensionality reduction methods for neurons with non-Poisson firing statistics, and show that they can be framed equivalently in likelihood-based or information-theoretic terms. Finally, we show how to overcome practical limitations on the number of stimulus dimensions that MID can estimate by constraining the form of the non-parametric nonlinearity in an LNP model. We illustrate these methods with simulations and data from primate visual cortex.

## Author Summary

A popular approach to the neural coding problem is to identify a low-dimensional linear projection of the stimulus space that preserves the aspects of the stimulus that affect a neuron’s probability of spiking. Previous work has focused on both information-theoretic and likelihood-based estimators for finding such projections. Here, we show that these two approaches are in fact equivalent. We show that maximally informative dimensions (MID), a popular information-theoretic method for dimensionality reduction, is identical to the maximum-likelihood estimator for a particular linear-nonlinear encoding model with Poisson spiking. One implication of this equivalence is that MID may not find the information-theoretically optimal stimulus projection when spiking is non-Poisson, which we illustrate with a few simple examples. Using these insights, we propose novel dimensionality-reduction methods that incorporate non-Poisson spiking, and suggest new parametrizations that allow for tractable estimation of high-dimensional subspaces.

## Introduction

The neural coding problem, an important topic in systems and computational neuroscience, concerns the probabilistic relationship between environmental stimuli and neural spike responses. Characterizing this relationship is difficult in general because of the high dimensionality of natural signals. A substantial literature therefore has focused on dimensionality reduction methods for identifying which stimuli affect a neuron’s probability of firing. The basic idea is that many neurons compute their responses in a low dimensional subspace, spanned by a small number of stimulus features. By identifying this subspace, we can more easily characterize the nonlinear mapping from stimulus features to spike responses [1–5].

Neural dimensionality-reduction methods can be coarsely divided into three classes: (1) moment-based estimators, such as spike-triggered average (STA) and covariance (STC) [1, 5–8]; (2) model-based estimators, which rely on explicit forward encoding models [9–16]; and (3) information and divergence-based estimators, which seek to reduce dimensionality using an information-theoretic cost function [17–22]. For all such methods, the goal is to find a set of linear filters, specified by the columns of a matrix *K*, such that the probability of response *r* given a stimulus **s** depends only on the linear projection of **s** onto these filters, i.e., *p*(*r*|**s**) ≈ *p*(*r*|*K* ^⊤^ **s**). Existing methods differ in computational complexity, modeling assumptions, and stimulus requirements. Typically, moment-based estimators have low computational cost but succeed only for restricted classes of stimulus distributions, whereas information-theoretic and likelihood-based estimators allow for arbitrary stimuli but have high computational cost. Previous work has established theoretical connections between moment-based and likelihood-based estimators [11, 14, 17, 19, 23], and between some classes of likelihood-based and information-theoretic estimators [14, 20, 21, 24].

Here we focus on maximally informative dimensions (MID), a well-known information-theoretic estimator introduced by Sharpee, Rust & Bialek [18]. We show that this estimator is formally identical to the maximum likelihood (ML) estimator for the parameters of a linear-nonlinear-Poisson (LNP) encoding model. Although previous work has demonstrated an asymptotic equivalence between these methods [20, 24, 25], we show that the correspondence is exact, regardless of time bin size or the amount of data. This equivalence follows from the fact that the plugin estimate for the single-spike information [26], the quantity that MID optimizes, is equal to a normalized Poisson log-likelihood.

The connection between the MID estimator and the LNP model makes clear that MID does not incorporate information carried by non-Poisson statistics of the response. We illustrate this shortcoming by showing that MID can fail to find information-maximizing filters for simulated neurons with binary or other non-Poisson spike count distributions. To overcome this limitation, we introduce new dimensionality-reduction estimators based on non-Poisson noise models, and show that they can be framed equivalently in information-theoretic or likelihood-based terms.

Finally, we show that a model-based perspective leads to strategies for overcoming a limitation of traditional MID, that it cannot tractably estimate more than two or three filters. The difficulty arises from the intractability of using histograms to estimate densities in high-dimensional subspaces. However, the single-spike information depends only on the ratio of densities, which is proportional to the nonlinearity in the LNP model. We show that by restricting the parametrization of this nonlinearity so that the number of parameters does not grow exponentially with the number of dimensions, we can obtain flexible yet computationally tractable estimators for models with many filters or dimensions.

## Results

### Background

#### Linear-nonlinear-Poisson (LNP) encoding model

**Fig 1:**
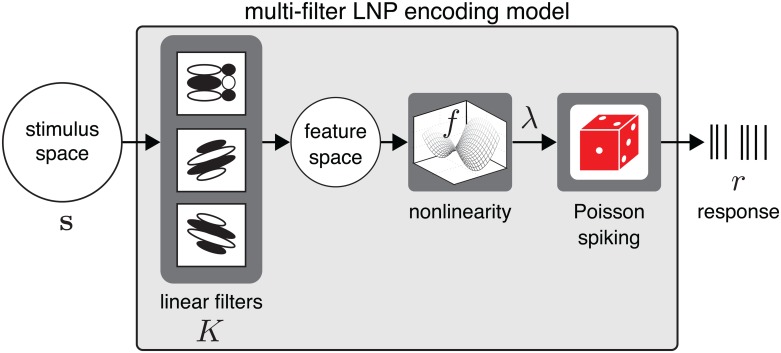
The linear-nonlinear-Poisson (LNP) encoding model formalizes the neural encoding process in terms of a cascade of three stages. First, the high-dimensional stimulus **s** projects onto bank of filters contained in the columns of a matrix *K*, resulting in a point in a low-dimensional neural feature space *K* ^⊤^ **s**. Second, an instantaneous nonlinear function *f* maps the filtered stimulus to an instantaneous spike rate *λ*. Third, spikes *r* are generated according to an inhomogeneous Poisson process.

Linear-nonlinear cascade models provide a useful framework for describing neural responses to high-dimensional stimuli. These models define the response in terms of a cascade of linear, nonlinear, and probabilistic spiking stages (see Fig. 1). The linear stage reduces the dimensionality by projecting the high-dimensional stimulus onto a set of linear filters, and a nonlinear function then converts the output of these filters to a non-negative spike rate.

Let *θ* = {*K*,*α*} denote the parameters of the LNP model, where *K* is a (tall, skinny) matrix whose columns contain the stimulus filters (for cases with a single filter, we will denote the filter with a vector **k** instead of the matrix *K*), and *α* are parameters governing the nonlinear function *f* from feature space to instantaneous spike rate. Under this model, the probability of a spike response *r* given stimulus **s** is governed by a Poisson distribution:

$$\begin{array}{ccc} \lambda & = & f(K^{⊤}s)\\ p(r|\lambda ) & = & \frac{1}{r!}{\left(\Delta \lambda \right)}^{r}e^{-\Delta \lambda }, \end{array}$$ (1)

where *λ* denotes the stimulus-driven spike rate (or “conditional intensity”) and Δ denotes a time bin size. The defining feature of a Poisson process is that responses in non-overlapping time bins are conditionally independent given the spike rate. In a discrete-time LNP model, the conditional probability of a dataset *D* = {(**s**_*t*_,*r*_*t*_)}, consisting of stimulus-response pairs indexed by *t* ∈ {1,…,*N*}, is the product of independent terms. The log-likelihood is therefore a sum over time bins:

$$\begin{array}{c} {ℒ}_{lnp}\left(\theta ;D\right)\;=\;\sum_{t=1}^{N}\log p\left(r_{t}|s_{t},\theta \right)\;=\;\sum_{t=1}^{N}\left(r_{t}\log\left(\Delta f\left(K^{⊤}s_{t}\right)\right)-\Delta f\left(K^{⊤}s_{t}\right)\right)\;-\;\left(\sum_{t=1}^{N}\log r_{t}!\right), \end{array}$$ (2)

where −(∑log*r*_*t*_!) is a constant that does not depend on *θ*. The ML estimate for *θ* is simply the maximizer of the log-likelihood: ${\hat{\theta }}_{ML}=\underset{\theta }{\text{arg}\;\text{max}}\;{ℒ}_{lnp}(\theta ;D)$.

#### Maximally informative dimensions (MID)

The maximally informative dimensions (MID) estimator seeks to find an informative low-dimensional projection of the stimulus by maximizing an information-theoretic quantity known as the single-spike information [26]. This quantity, which we denote *I*_*ss*_, is the the average information that the time of a single spike (considered independently of other spikes) carries about the stimulus

Although first introduced as a quantity that can be computed from the peri-stimulus time histogram (PSTH) measured in response to a repeated stimulus, the single-spike information can also be expressed as the Kullback-Leibler (KL) divergence between two distributions over the stimulus (see [26], appendix B):

$$\begin{array}{c} I_{ss}=\int p\left(s|spike\right)\log\frac{p(s|spike)}{p(s)}ds=D_{KL\;}\left(p(s|spike)\;|\;|\;p(s)\right), \end{array}$$ (3)

where *p*(**s**) denotes the marginal or “raw” distribution over stimuli, and *p*(**s**|*spike*) is the distribution over stimuli conditioned on observing a spike, also known as the “spike-triggered” stimulus distribution. Note that *p*(**s**|*spike*) is *not* the same as *p*(**s**|*r* = 1), the distribution of stimuli conditioned on a spike count of *r* = 1, since a stimulus that elicits two spikes will contribute twice as much to the spike-triggered distribution as a stimulus that elicits only one spike.

**Fig 2:**
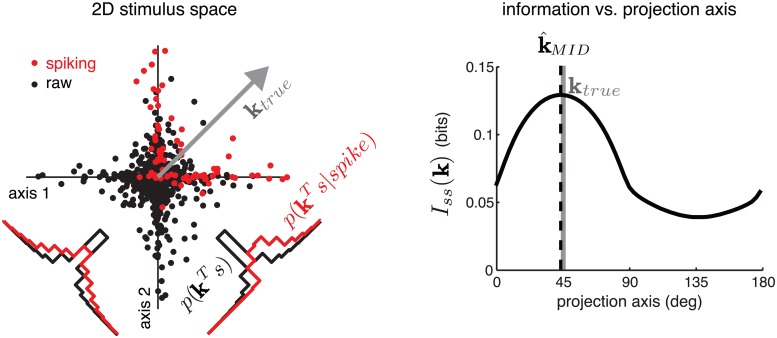
Geometric illustration of maximally-informative-dimensions (MID). **Left:** A two-dimensional stimulus space, with points indicating the location of raw stimuli (black) and spike-eliciting stimuli (red). For this simulated example, the probability of spiking depended only on the projection onto a filter **k**_*true*_, oriented at 45^∘^. Histograms (inset) show the one-dimensional distributions of raw (black) and spike-triggered stimuli (red) projected onto **k**_*true*_ (lower right) and its orthogonal complement (lower left). **Right:** Estimated single-spike information captured by a 1D subspace, as a function of the axis of projection. The MID estimate ${\hat{\text{k}}}_{MID}$ (dotted) corresponds to the axis maximizing single-spike information, which converges asymptotically to **k**_*true*_ with dataset size.

The MID estimator [18] seeks to find the linear projection that preserves maximal single-spike information:

$$\begin{array}{c} I_{ss}\left(K\right)=D_{KL\;}\left(p\left(K^{⊤}s|spike\right)\;|\;|\;p\left(K^{⊤}s\right)\right), \end{array}$$ (4)

where *p*(*K* ^⊤^ **s**) and *p*(*K* ^⊤^ **s**|*spike*) are the raw and spike-triggered stimulus distributions projected onto the subspace defined by the columns of *K*, respectively. In practice, the MID estimator maximizes an estimate of the projected single-spike information:

$$\begin{array}{c} {\hat{K}}_{{}_{MID}}=\text{arg}\underset{K}{\text{max}}\;{\hat{I}}_{ss}\left(K\right), \end{array}$$ (5)

where *Î*_*ss*_(*K*) denotes an empirical estimate of *I*_*ss*_(*K*). The columns of ${\hat{K}}_{MID}$ can be conceived as “directions” or “axes” in stimulus space that are most informative about a neuron’s probability of spiking, as quantified by single-spike information. Fig. 2 shows a simulated example illustrating the MID estimate for a single linear filter in a two-dimensional stimulus space.

### Equivalence of MID and maximum-likelihood LNP

Previous work has shown that MID converges asymptotically to the maximum-likelihood (ML) estimator for an LNP model in the limit of small time bins [20, 24]. Here we present a stronger result, showing that the equivalence is not merely asymptotic. We show that standard MID, using histogram-based estimators for raw and spike-triggered stimulus densities *p*(**s**) and *p*(**s**|*spike*), is exactly the ML estimator for the parameters of an LNP model, regardless of spike rate, the time bins used to count spikes, or the amount of data.

The standard implementation of MID [18, 20] uses histograms to estimate the projected stimulus densities *p*(*K* ^⊤^ **s**) and *p*(*K* ^⊤^ **s**|*spike*). These density estimates are then used to compute *Î*_*ss*_(*K*), the plug-in estimate of single-spike information in a subspace defined by *K* (Equation 4). We will now unpack the details of this estimate in order to show its relationship to the LNP model log-likelihood.

Let {*B*_1_, …, *B*_*m*_} denote a group of sets (“histogram bins”) that partition the range of the projected stimuli *K* ^⊤^ **s**. In the one-dimensional case, we typically choose these sets to be intervals *B*_*i*_ = [*b*_*i*−1_,*b*_*i*_), defined by bin edges {*b*_0_, …, *b*_*m*_}, where *b*_0_ = −∞ and *b*_*m*_ = +∞. Then let $\hat{\text{p}}=({\hat{p}}_{1},\ldots ,{\hat{p}}_{m})$ and $\hat{\text{q}}=({\hat{q}}_{1},\ldots {\hat{q}}_{m})$ denote histogram-based estimates of *p*(*K* ^⊤^ **s**) and *p*(*K* ^⊤^ **s**|*spike*), respectively, given by:

$$\begin{array}{cc} {\hat{p}}_{i}\; & =\;\frac{\#\text{stimuli in}\;B_{i}}{\#\text{stimuli}}\;=\;\frac{1}{N}\sum_{t=1}^{N}1_{B_{i}}\left(x_{t}\right)\\ {\hat{q}}_{i}\; & =\;\frac{\#\text{stimuli in}\;B_{i}|\mathrm{spike}}{\#\mathrm{spikes}}\;=\;\frac{1}{n_{sp}}\sum_{t=1}^{N}1_{B_{i}}\left(x_{t}\right)r_{t}, \end{array}$$ (6)

where **x**_*t*_ = *K* ^⊤^ **s**_*t*_ denotes the linear projection of the stimulus **s**_*t*_, $n_{sp}=\sum_{t=1}^{N}r_{t}$ is the total number of spikes, and **1**_*B*_*i*__(⋅) is the indicator function for the set *B*_*i*_, defined as:

$$\begin{array}{c} 1_{B_{i}}\left(x\right)=\left\{\begin{array}{cc} 1, & x\in B_{i}\\ 0, & x\notin B_{i} \end{array}\right. \end{array}$$ (7)

The estimates $\hat{\text{p}}$ and $\hat{\text{q}}$ are also known as the “plug-in” estimates, and correspond to maximum likelihood estimates for the densities in question. These estimates give us a plug-in estimate for projected single-spike information:

$$\begin{array}{c} {\hat{I}}_{ss}\;=\;\sum_{i=1}^{m}{\hat{q}}_{i}\log\frac{{\hat{q}}_{i}}{{\hat{p}}_{i}}\;=\;\frac{1}{n_{sp}}\sum_{i=1}^{m}\sum_{t=1}^{N}1_{B_{i}}\left(x_{t}\right)r_{t}\log\frac{{\hat{q}}_{i}}{{\hat{p}}_{i}}\;=\;\frac{1}{n_{sp}}\sum_{t=1}^{N}r_{t}\log\hat{g}\left(x_{t}\right) \end{array}$$ (8)

where the function *ĝ*(**x**) denotes the ratio of density estimates:

$$\begin{array}{c} \hat{g}\left(x\right)≜\sum_{i=1}^{m}1_{B_{i}}\left(x\right)\frac{{\hat{q}}_{i}}{{\hat{p}}_{i}}. \end{array}$$ (9)

Note that *ĝ*(**x**) is a piece-wise constant function that takes the value *q̂*_*i*_/*p̂*_*i*_ over the *i*th histogram bin *B*_*i*_.

Now, consider an LNP model in which the nonlinearity *f* is parametrized as a piece-wise constant function, taking the value *f*_*i*_ over histogram bin *B*_*i*_. Given a projection matrix *K*, the ML estimate for the parameter vector *α* = (*f*_1_, …, *f*_*m*_) is the average number of spikes per stimulus in each histogram bin, divided by time bin width Δ, that is:

$$\begin{array}{c} {\hat{f}}_{i}=\frac{1}{\Delta }\cdot \frac{\sum_{t=1}^{N}1_{B_{i}}\left(x_{t}\right)r_{t}}{\sum_{t=1}^{N}1_{B_{i}}\left(x_{t}\right)}=\left(\;\frac{n_{sp}}{N\Delta }\;\right)\frac{{\hat{q}}_{i}}{{\hat{p}}_{i}}. \end{array}$$ (10)

Note that functions $\hat{f}$ and $\hat{g}$ are related by $\hat{f}(\text{x})=\left(\frac{n_{sp}}{N\Delta }\right)\hat{g}(\text{x})$ and that the sum $\sum_{t=1}^{N}\hat{f}({\text{x}}_{t})\Delta =n_{sp}$. We can therefore rewrite the LNP model log-likelihood (Equation 2):

$$\begin{array}{ccc} {ℒ}_{lnp}\left(\theta ;D\right) & = & \sum_{t=1}^{N}r_{t}\log\left(\frac{n_{sp}}{N}\hat{g}\left(x_{t}\right)\right)\;-\;n_{sp}-\sum_{t=1}^{N}\log r_{t}!\\ & = & \sum_{t=1}^{N}r_{t}\log\hat{g}\left(x_{t}\right)\;+\;n_{sp}\left(\log\frac{n_{sp}}{N}-1\right)-\sum_{t=1}^{N}\log r_{t}! \end{array}$$ (11)

This allows us to directly relate the empirical single-spike information (Equation 8) with the LNP model log-likelihood, normalized by the spike count as follows:

$$\begin{array}{ccc} {\hat{I}}_{ss}\left(K\right) & = & \frac{1}{n_{sp}}{ℒ}_{lnp}\left(\theta ;D\right)\;-\;\frac{1}{n_{sp}}\left[n_{sp}\log\frac{n_{sp}}{N}-n_{sp}-\sum \log r_{t}!\right] \end{array}$$ (12)

$$\begin{array}{ccc} & = & \frac{1}{n_{sp}}{ℒ}_{lnp}\left(\theta ;D\right)\;-\;\frac{1}{n_{sp}}{ℒ}_{lnp}\left(\theta_{0},D\right) \end{array}$$ (13)

where ℒ_*lnp*_(*θ*_0_,*D*) denotes the Poisson log-likelihood under a “null” model in which spike rate does not depend on the stimulus, but takes constant rate $\lambda_{0}=\frac{n_{sp}}{N\Delta }$ across the entire stimulus space. In fact, the quantity −ℒ_*lnp*_(*θ*_0_,*D*) can be considered an estimate for the marginal entropy of the response distribution, H(r)=−∑p(r)logp(r), since it is the average log-probability of the response under a Poisson model, independent of the stimulus. This makes it clear that the single-spike information *I*_*ss*_ can be equally regarded as “LNP information”.

Empirical single-spike information is therefore equal to LNP model log-likelihood per spike, plus a constant that does not depend on model parameters. This equality holds independent of time bin size Δ, the number of samples *N* and the number of spikes *n*_*sp*_. From this relationship, it is clear that the linear projection *K* that maximizes *Î*_*ss*_ also maximizes the LNP log-likelihood ℒ_*lnp*_(*θ*;*D*), meaning that the MID estimate is the same as an ML estimate for the filters in an LNP model:

$$\begin{array}{c} {\hat{K}}_{MID}={\hat{K}}_{ML}. \end{array}$$ (14)

Moreover, the histogram-based estimates of the raw and spike-triggered stimulus densities $\hat{\text{p}}$ and $\hat{\text{q}}$, which are used for computing the empirical single-spike information *Î*_*ss*_, correspond to a particular parametrization of the LNP model nonlinearity *f* as a piece-wise constant function over histogram bins. The ratio of these plug-in estimates gives rise to the ML estimate for *f*. MID is thus formally equivalent to an ML estimator for both the linear filters and the nonlinearity of an LNP model.

**Fig 3:**
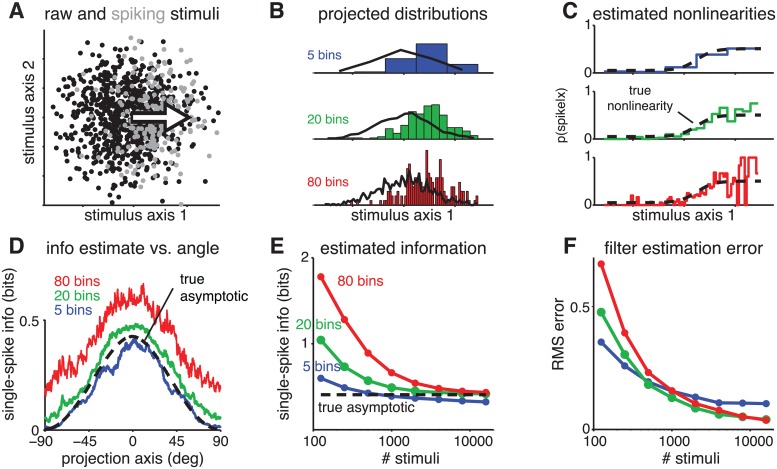
Effects of the number of histogram bins on empirical single-spike information and MID performance. **(A)** Scatter plot of raw stimuli (black) and spike-triggered stimuli (gray) from a simulated experiment using two-dimensional stimuli to drive a linear-nonlinear-Bernoulli neuron with sigmoidal nonlinearity. Arrow indicates the direction of the true filter **k**. **(B)** Plug-In estimates of *p*(**k** ^⊤^ **s**|*spike*), the spike-triggered stimulus distribution along the true filter axis, from 1000 stimuli and 200 spikes, using 5 (blue), 20 (green) or 80 (red) histogram bins. Black traces show estimates of raw distribution *p*(**k** ^⊤^ **s**) along the same axis. **(C)** True nonlinearity (black) and ML estimates of the nonlinearity (derived from the ratio of the density estimates shown in B). Roughness of the 80-bin estimate (red) arises from undersampling, or (equivalently) overfitting of the nonlinearity. **(D)** Empirical single-spike information vs. direction, calculated using 5, 20 or 80 histogram bins. Note that the 80-bin model overestimates the true asymptotic single-spike information at the peak by a factor of more than 1.5. **(E)** Convergence of empirical single-spike information along the true filter axis as a function of sample size. With small amounts of data, all three models overfit, leading to upward bias in estimated information. For large amounts of data, the 5-bin model underfits and therefore under-estimates information, since it lacks the smoothness to adequately describe the shape of the sigmoidal nonlinearity. **(F)** Filter error as a function of the number of stimuli, showing that the optimal number of histogram bins depends on the amount of data.

Previous literature has not emphasized that the MID estimator implicitly provides an estimate of the LNP model nonlinearity, or that the number of histogram bins corresponds to the number of parameters governing the nonlinearity. Selecting the number of parameters for the nonlinearity is important both for accurately estimating single-spike information from finite data and for successfully finding the most informative filter or filters. Fig. 3 illustrates this point using data from a simulated neuron with a single filter in a two-dimensional stimulus space. For small datasets, the MID estimate computed with many histogram bins (i.e., many parameters for the nonlinearity) substantially overestimates the true *I*_*ss*_ and yields large errors in the filter estimate. Even with 1000 stimuli and 200 spikes, a 20-bin histogram gives substantial upward bias in the estimate of single-spike information (Fig. 3D). Parametrization of the nonlinearity is therefore an important problem that should be addressed explicitly when using MID, e.g., by cross-validation or other model selection methods.

### Models with Bernoulli spiking

Under the discrete-time inhomogeneous Poisson model considered above, spikes are modeled as conditionally independent given the stimulus, and the spike count in a discrete time bin has a Poisson distribution. However, real spike trains may exhibit more or less variability than a Poisson process [27]. In particular, the Poisson assumption breaks down when the time bin in which the data are analyzed approaches the length of the refractory period, since in that case each bin can contain at most one spike. In that case, a Bernoulli model provides a more accurate description of neural data, since it allows only 0 or 1 spike per bin. The Bernoulli and discrete-time Poisson models approach the same limiting Poisson process as the bin size (and single-bin spike probability) approaches zero while the average spike rate remains constant. However, as long as single-bin spike probabilities are above zero, the two models differ.

Here we show that the standard “Poisson” MID estimator does not necessarily maximize information between stimulus and response when spiking is non-Poisson. That is, if the spike count *r* given stimulus **s** is not a Poisson random variable, then MID does not necessarily find the subspace preserving maximal information between stimulus and response. To show this, we derive the mutual information between the stimulus and a Bernoulli distributed spike count, and show that this quantity is closely related to the log-likelihood under a linear-nonlinear-Bernoulli encoding model.

#### Linear-nonlinear-Bernoulli (LNB) model

We can define the linear-nonlinear-Bernoulli (LNB) model by analogy to the LNP model, but with Bernoulli instead of Poisson spiking. The parameters *θ* = {*K*,*α*} consist of a matrix *K* that determines a linear projection of the stimulus space, and a set of parameters *α* that govern the nonlinearity *f*. Here, the output of *f* is spike probability *λ* in the range [0, 1]. The probability of a spike response *r* ∈ {0,1} given stimulus **s** is governed by a Bernoulli distribution. We can express this model as

$$\begin{array}{ccc} \lambda & = & f(K^{⊤}s) \end{array}$$ (15)

$$\begin{array}{ccc} p(r|\lambda ) & = & \lambda^{r}{\left(1-\lambda \right)}^{1-r}, \end{array}$$ (16)

and the log-likelihood for a dataset *D* = {(**s**_*t*_,*r*_*t*_)} is

$$\begin{array}{c} {ℒ}_{lnb}\left(\theta ;D\right)\;=\;\sum_{t=1}^{N}\left(r_{t}\log\left(f\left(K^{⊤}s_{t}\right)\right)-\left(1-r_{t}\right)\log\left(1-f\left(K^{⊤}s_{t}\right)\right)\right). \end{array}$$ (17)

If *K* has a single filter and the nonlinearity is restricted to be a logistic function, *f*(*x*) = 1/(1+exp(−*x*)), this reduces to the logistic regression model. Note that the spike probability *λ* is analogous to the single-bin Poisson rate *λ*Δ from the LNP model (Equation 1), and the two models become identical in the small-bin limit where the probability of spiking *p*(*r* = 1) goes to zero [24, 26].

#### Bernoulli information

We can derive an equivalent dimensionality-reduction estimator in information-theoretic terms. The mutual information between the projected stimulus **x** = *K* ^⊤^ **s** and a Bernoulli spike response *r* ∈ {0,1} is given by:

$$\begin{array}{cc} I\left(x,r\right) & =\;H\left(x\right)-H(x|r)\\ & =\;-\int^{\text{​}}dxp\left(x\right)\log p\left(x\right)\;+\;\underset{j\in \left\{0,1\right\}}{\sum }\;p\left(r=j\right)\int^{\text{​}}dxp(x|r=j)\log p(x|r=j)\\ & =\;\underset{j\in \left\{0,1\right\}}{\sum }\;p\left(r=j\right)\int^{\text{​}}dxp(x|r=j)\log\frac{p(x|r=j)}{p\left(x\right)}\\ & =\;\underset{j\in \left\{0,1\right\}}{\sum }\;p\left(r=j\right)D_{KL\text{​}}\left(pxr=j\left|\text{​}\right|p\left(x\right)\right). \end{array}$$ (18)

If we normalize by the probability of observing a spike, we obtain a quantity with units of bits-per-spike that can be directly compared to single-spike information. We refer to this as the Bernoulli information:

$$\begin{array}{c} I_{Ber}=\frac{1}{p(r=1)}I\left(x,r\right)=I_{0}+I_{ss} \end{array}$$ (19)

where $I_{0}=\frac{p(r=0)}{p(r=1)}D_{KL\;}(p(\text{x}|r=0)\;|\;|\;p(\text{x}))$ is the information (per spike) carried by silences, and *I*_*ss*_ is the single-spike information (Equation 4). Thus, where single-spike information quantifies the information conveyed by each spike alone (no matter how many spikes might co-occur in the same time bin) but neglects the information conveyed by silences, the Bernoulli information reflects the information conveyed by both spikes and silences.

Let *Î*_*Ber*_ = *Î*_0_+*Î*_*ss*_ denote the empirical or plug-in estimate of the Bernoulli information, where *Î*_*ss*_ is the empirical single-spike information (Equation 8), and *Î*_0_ is a plug-in estimate of the KL divergence between *p*(**x**|*r* = 0) and *p*(**x**), weighted by (*N*−*n*_*sp*_)/*n*_*sp*_, the ratio of the number of silences to the number of spikes. It is straightforward to show that empirical Bernoulli information equals the LNB model log-likelihood per spike plus a constant:

$$\begin{array}{c} {\hat{I}}_{Ber}=\frac{1}{n_{sp}}{ℒ}_{lnb}+\frac{1}{\bar{r}}\hat{H}\left[r\right] \end{array}$$ (20)

where $\bar{r}=\frac{n_{sp}}{N}$ denotes mean spike count per bin and $\hat{H}[r]=-\frac{n_{sp}}{N}\log\frac{n_{sp}}{N}-\frac{N-n_{sp}}{N}\log\frac{N-n_{sp}}{N}$ is the plug-in estimate for the marginal response entropy. Because the second term is independent of *θ*, the maximum of the empirical Bernoulli information is identical to the maximum of the LNB model likelihood, meaning that once again, we have an exact equivalence between likelihood-based and information-based estimators.

#### Failure modes for MID under Bernoulli spiking

The empirical Bernoulli information is strictly greater than the estimated single-spike (or “Poisson”) information for a binary spike train that is not all zeros or ones, since *Î*_0_ > 0 and these spike absences are neglected by the single-spike information measure. Only in the limit of infinitesimal time bins, where *p*(*r* = 1) → 0, does *Î*_*Ber*_ converge to *Î*_*ss*_[24, 26]. As a result, standard MID can fail to identify the most informative subspace when applied to a neuron with Bernoulli spiking. We illustrate this phenomenon with two (admittedly toy) simulated examples. For both examples, we compute the standard MID estimate ${\hat{\text{k}}}_{MID}$ by maximizing *Î*_*ss*_, and the LNB filter estimate ${\hat{\text{k}}}_{Ber}$ which maximizes the LNB likelihood Squashed hats.

**Fig 4:**
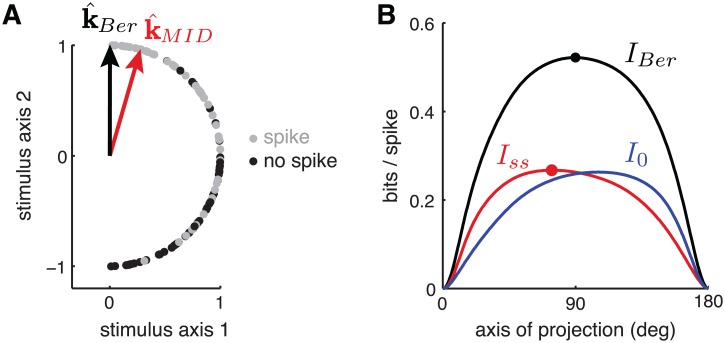
Illustration of MID failure mode due to non-Poisson spiking. **(A)** Stimuli were drawn uniformly on the unit half-circle, *θ* ∼ Unif(−*π*/2,*π*/2). The simulated neuron had Bernoulli (i.e., binary) spiking, where the probability of a spike increased linearly from 0 to 1 as *θ* varied from -*π*/2 to *π*/2, that is: *p*(*spike*|*θ*) = *θ*/*π*+1/2. Stimuli eliciting “spike” and “no-spike” are indicated by gray and black circles, respectively. For this neuron, the most informative one-dimensional linear projection corresponds to the vertical axis (${\hat{\text{k}}}_{Ber}$), but the MID estimator (${\hat{\text{k}}}_{MID}$) exhibits a 16^∘^ clockwise bias. **(B)** Information from spikes (black), silences (gray), and both (red), as a function of projection angle. The peak of the Bernoulli information (which defines ${\hat{\text{k}}}_{Ber}$) lies close to *π*/2, while the peak of single-spike information (which defines ${\hat{\text{k}}}_{MID}$) exhibits the clockwise bias shown in A. Note that ${\hat{\text{k}}}_{MID}$ does not converge to the optimal direction even in the limit of infinite data, due to its lack of sensitivity to information from silences. Although this figure is framed in an information-theoretic sense, equations (19) and (20) detail the equivalence between *I*_*Ber*_ and ℒ_*lnb*_, so that this figure can be viewed from either an information-theoretic or likelihood-based perspective.

The first example (Fig. 4) uses stimuli uniformly distributed on the right half of the unit circle. The Bernoulli spike probability *λ* increases linearly as a function of stimulus angle: *λ* = (**s**−*π*/2)/*π*, for **s** ∈ (−*π*/2,*π*/2]. For this neuron, the most informative 1D axis is the vertical axis, which is closely matched by the estimate ${\hat{\text{k}}}_{Ber}$. By contrast, ${\hat{\text{k}}}_{MID}$ exhibits a substantial clockwise bias, resulting from its failure to take into account the information from silences (which are more informative when spike rate is high). Fig. 4B shows the breakdown of total Bernoulli information into *Î*_*ss*_ (spikes) and *Î*_0_ (silences) as a function of projection angle, which illustrates the relative biases of the two quantities.

**Fig 5:**
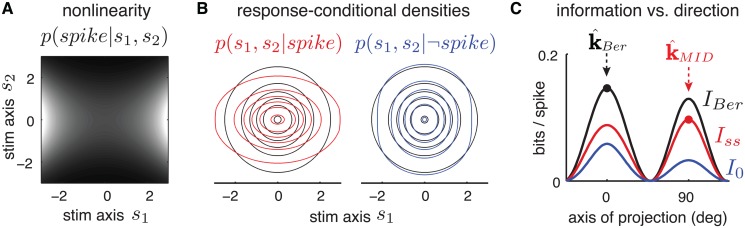
A second example Bernoulli neuron for which ${\hat{k}}_{MID}$ fails to identify the most-informative one-dimensional subspace. The stimulus space has two dimensions, denoted *s*_1_ and *s*_2_, and stimuli were drawn *iid* from a standard Gaussian 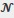
(0,1). **(A)** The nonlinearity *f*(*s*_1_,*s*_2_) = *p*(*spike*|*s*_1_,*s*_2_) is excitatory in *s*_1_ and suppressive in *s*_2_; brighter intensity indicates higher spike probability. **(B)** Contour plot of the stimulus-conditional densities given the two possible responses: “spike” (red) or “no-spike” (blue), along with the raw stimulus distribution (black). **(C)** Information carried by silences (*I*_0_), single spikes (*I*_*ss*_), and total Bernoulli information (*I*_*Ber*_ = *I*_0_+*I*_*ss*_) as a function of subspace orientation. The MID estimate ${\hat{k}}_{MID}=90^{\circ }$ is the maximum of *I*_*ss*_, but the total Bernoulli information is in fact 13% higher at ${\hat{\text{k}}}_{Ber}=0^{\circ }$ due to the incorporation of no-spike information. Although both stimulus axes are clearly relevant to the neuron, MID identifies the less informative one. As with the previous figure, equations (19) and (20) detail the equivalence between *I*_*Ber*_ and ℒ_*lnb*_, so that this figure can be viewed from either an information-theoretic or likelihood-based perspective.

A second example (Fig. 5) uses stimuli drawn from a standard bivariate Gaussian (0 mean and identity covariance), in which standard MID makes a 90 deg error in identifying the most informative one-dimensional subspace. The neuron’s nonlinearity (Fig. 5A) is excitatory in stimulus axis *s*_1_ and suppressive in stimulus axis *s*_2_ (indicating that a large projection onto *s*_1_ increases spike probability, while a large projection onto *s*_2_ decreases spike probability). For this neuron, both stimulus axes are clearly informative, but the (suppressive, vertical) axis *s*_2_ carries 13% more information than the (excitatory, horizontal) axis *s*_1_. However, the standard MID estimator identifies *s*_1_ as the most informative axis (Fig. 5C), due once again to the failure to account for the information carried by silences.

These artificial examples were designed to emphasize the information carried by missing spikes, and we do not expect such stark differences between Bernoulli and Poisson estimators to arise in typical neural data. However, it is clear that the assumption of Poisson firing can lead the standard MID estimator to make mistakes when spiking is actually Bernoulli. In general, we suggest that the question of which estimator performs better is an empirical one, and depends on which model describes the true spiking process more accurately.

#### Quantifying MID information loss for binary spike trains

In the limit of infinitesimal time bins, the information carried by silences goes to zero, and the plug-in estimates for Bernoulli and single-spike information converge: *I*_0_ → 0 and *Î*_*Ber*_ → *Î*_*ss*_. However, for finite time bins, the Bernoulli information can substantially exceed single-spike information. In the previous section, we showed that this mismatch can lead to errors in subspace identification. Here we derive a lower bound on the information lost due to the neglect of *I*_0_, the information (per spike) carried by silences, as a function of marginal probability of a spike, *p*(*r* = 1).

In the limit of rare spiking, *p*(*r* = 1) → 0, we find that:

$$\begin{array}{c} \frac{I_{0}}{I_{Ber}}=\frac{I_{0}}{I_{0}+I_{ss}}\geq \frac{p(r=1)}{2}. \end{array}$$ (21)

**Fig 6:**
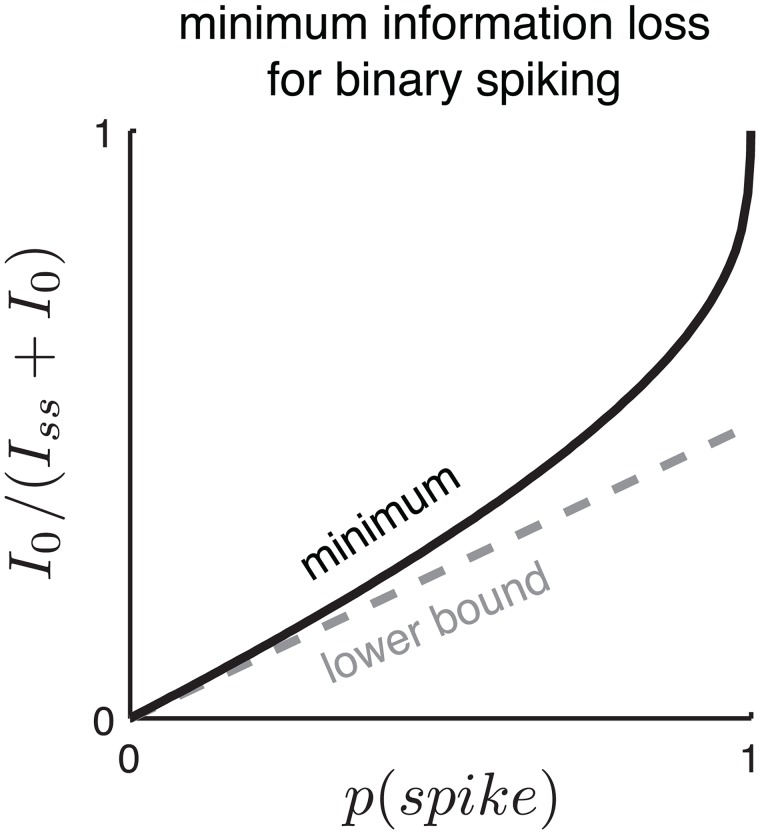
Lower bound on the fraction of total information neglected by MID for a Bernoulli neuron, as a function of the marginal spike probability *p*(*spike*) = *p*(*r* = 1), for the special case of a binary stimulus. Information loss is quantified as the ratio *I*_0_/(*I*_0_+*I*_*ss*_), the information due to no-spike events, *I*_0_, divided by the total information due to spikes and silences, *I*_0_+*I*_*ss*_. The dashed gray line shows the lower bound derived in the limit *p*(*spike*) → 0. The solid black line shows the actual minimum achieved for binary stimuli *s* ∈ {0,1} with *p*(*s* = 1) = *q*, computed via a numerical search over the parameter *q* ∈ [0, 1] for each value of *p*(*spike*). The lower bound is substantially loose for *p*(*spike*) > 0, since as *p*(*spike*) → 1, the fraction of information due to silences goes to 1.

The fraction of lost information is at least half the marginal spike probability. Thus, for example, if 20% of the bins in a binary spike train contain a spike, the standard MID estimator will necessarily neglect at least 10% of the total mutual information. We show this bound holds in the asymptotic limit of small *p*(*r* = 1) (see Methods for details), but conjecture that it holds for all *p*(*r* = 1). The bound is tight in the Poisson limit, *p*(*r* = 1) → 0, but is substantially loose in the limit where spiking is common *p*(*r* = 1) → 1, in which all information is carried by silences. Fig. 6 shows our bound compared to the actual (numerical) lower bound for an example with a binary stimulus.

### Models with arbitrary spike count distributions

For neural responses binned at the stimulus refresh rate (e.g., 100 Hz), it is not uncommon to observe multiple spikes in a single bin. For the general case, then, we must consider an arbitrary distribution over counts conditioned on a stimulus. As we will see, maximizing the mutual information based on histogram estimators is once again equivalent to maximizing the likelihood of an LN model with piece-wise constant mappings from the linear stimulus projection to count probabilities.

#### Linear-nonlinear-count (LNC) model

Suppose that a neuron responds to a stimulus **s** with a spike count *r* ∈ {0,…,*r*_max_}, where *r*_max_ is the maximum possible number of spikes within the time bin (constrained by the refractory period or firing rate saturation). The linear-nonlinear-count (LNC) model, which includes LNB as a special case, is defined by a linear dimensionality reduction matrix *K* and a set of nonlinear functions {*f* ^(0)^ …, *f* ^(*r*_max_)^} that map the projected stimulus to the probability of observing {0, …, *r*_max_} spikes, respectively. We can write the probability of a spike response *r* given projected stimulus **x** = *K* ^⊤^ **s** as:

$$\begin{array}{ccc} \lambda^{\left(j\right)} & = & f^{\left(j\right)}\left(x\right),\;\mathrm{for}\;j=0,\ldots ,r_{\text{max}}\\ p(r=j|\lambda^{\left(j\right)}) & = & \lambda^{\left(j\right)}. \end{array}$$ (22)

Note that there is a constraint on the functions *f* requiring that ∑_*j*_ *f* ^(*j*)^(**x**) = 1,∀**x**, since the probabilities over possible counts must add to 1 for each stimulus.

The LNC model log-likelihood for parameters *θ* = (*K*, *α* ^(0)^, … *α* ^(*r*_max_)^) given data *D* = {(**s**_*t*_,*r*_*t*_)} can be written:

$$\begin{array}{c} {ℒ}_{lnc}\left(\theta ;D\right)\;=\;\sum_{t=1}^{N}\sum_{j=0}^{r_{\text{max}}}\left(1_{j}\left(r_{t}\right)\;\log\left(f^{\left(j\right)}\left(K^{⊤}s_{t}\right)\right)\right), \end{array}$$ (23)

where **1**_*j*_(*r*_*t*_) is an indicator function selecting time bins *t* in which the spike count is *j*. As before, we consider the case where *f* ^(*j*)^ takes a constant value in each of *m* histogram bins {*B*_*i*_}, so that the parameters are just those constant values: $\alpha^{\left(j\right)}=(f_{0}^{\left(j\right)},\ldots ,f_{m}^{\left(j\right)})$. The maximum-likelihood estimates for the values can be given in terms of the histogram probabilities:

$$\begin{array}{c} {\hat{f}}_{i}^{\left(j\right)}\;=\;\frac{n_{i}^{\left(j\right)}}{n_{i}}\;=\;\frac{{\hat{q}}_{i}^{\left(j\right)}}{{\hat{p}}_{i}}\frac{N^{\left(j\right)}}{N}\;. \end{array}$$ (24)

where *n*_*i*_ is the number of stimuli in bin *B*_*i*_, $n_{i}^{\left(j\right)}$ is the number of stimuli in bin *B*_*i*_ that elicited *j* spikes, *N* ^(*j*)^ is the number of stimuli in all bins that elicited *j* spikes, and *N* is the total number of stimuli. The histogram fractions of the projected raw spike counts *p̂*_*i*_ are defined as in Equation 6, with the *j*-spike conditioned histograms defined analogously:

$$\begin{array}{cc} {\hat{q}}_{i}^{\left(j\right)} & =\frac{1}{N^{\left(j\right)}}\sum_{t}1_{B_{i}}\left(x_{t}\right)\;1_{j}\left(r_{t}\right)\;=\;\frac{n_{i}^{\left(j\right)}}{N^{\left(j\right)}}\;, \end{array}$$ (25)

Thus, the log-likelihood for projection matrix *K*, having already maximized with respect to the nonlinearities by using their plug-in estimates, is

$$\begin{array}{cc} {ℒ}_{lnc}\left(K;D\right) & =\sum_{t=1}^{N}\sum_{j=0}^{r_{\text{max}}}\left(1_{j}\left(r_{t}\right)\;\log\left({\hat{f}}^{\left(j\right)}\left(K^{⊤}s_{t}\right)\right)\right) \end{array}$$ (26)

$$=\overset{N}{\underset{t=1}{\sum }}\overset{r_{\text{max}}}{\underset{j=0}{\sum }}\overset{m}{\underset{i=1}{\sum }}\left(1_{j}\left(r_{t}\right)1_{B_{i}}\left(K^{⊤}s_{t}\right)\text{log}\left({\hat{f}}_{i}^{\left(j\right)}\right)\right)$$ (27)

$$\begin{array}{cc} & =\sum_{j=0}^{r_{\text{max}}}\sum_{i=1}^{m}\left(n_{i}^{\left(j\right)}\log\frac{n_{i}^{\left(j\right)}}{n_{i}}\right) \end{array}$$ (28)

$$\begin{array}{cc} & =\sum_{j=0}^{r_{\text{max}}}\sum_{i=1}^{m}\left(N^{\left(j\right)}{\hat{q}}_{i}^{\left(j\right)}\log\left(\frac{{\hat{q}}_{i}^{\left(j\right)}}{{\hat{p}}_{i}}\frac{N^{\left(j\right)}}{N}\right)\right) \end{array}$$ (29)

$$\begin{array}{cc} & =\sum_{j=0}^{r_{\text{max}}}\sum_{i=1}^{m}\left(N^{\left(j\right)}{\hat{q}}_{i}^{\left(j\right)}\log\left(\frac{{\hat{q}}_{i}^{\left(j\right)}}{{\hat{p}}_{i}}\right)\right)+\sum_{j=0}^{r_{\text{max}}}\left(N^{\left(j\right)}\log\left(\frac{N^{\left(j\right)}}{N}\right)\right)\;. \end{array}$$ (30)

#### Information in spike counts

If the binned spike-counts *r*_*t*_ measured in response to stimuli **s**_*t*_ are not Poisson distributed, the projection matrix *K* which maximizes the mutual information between *K* ^⊤^ **s** and *r* can be found as follows. Recalling that *r*_max_ is the maximal spike count possible in the time bin and writing **x** = *K* ^⊤^ **s**, we have:

$$\begin{array}{cc} I(x,r) & =H(x)-H(x|r) \end{array}$$ (31)

$$\begin{array}{cc} & =-\int dx\;p\left(x\right)\log p\left(x\right)+\sum_{j=0}^{r_{\text{max}}}p\left(r=j\right)\int dx\;p\left(x|r=j\right)\log p\left(x|r=j\right) \end{array}$$ (32)

$$\begin{array}{cc} & =\sum_{j=0}^{r_{\text{max}}}p\left(r=j\right)\int dx\;p\left(x|r=j\right)\log\frac{p(x|r=j)}{p(x)} \end{array}$$ (33)

$$\begin{array}{cc} & =\sum_{j=0}^{r_{\text{max}}}p\left(r=j\right)D_{KL\;}\left(p(x|r=j)\;|\;|\;p(x)\right)\;. \end{array}$$ (34)

To ease comparison with the single-spike information, which is measured in bits per spike, we normalize the mutual information by the mean spike count to obtain:

$$\begin{array}{c} I_{count}=\frac{1}{\bar{r}}I\left(x,r\right)=I_{0}+I_{1}+\cdots +I_{r_{\text{max}}} \end{array}$$ (35)

where *r̄* = ∑_*t*_ *r*_*t*_/*N* is the mean spike count, and $I_{j}=\frac{p(r=j)}{\bar{r}}D_{KL\;}(p(\text{x}|r=j)\;|\;|\;p(\text{x}))$ is the normalized information carried by the *j*-spike responses. Note that *I*_1_, the information carried by single-spike responses, is *not* the same as the single-spike information *I*_*ss*_, since the latter combines information from all responses with 1 or more spikes, by assuming that each spike is conditionally independent of all other spikes.

We can estimate the mutual information from data using a histogram based plug-in estimator:

$$\begin{array}{c} {\hat{I}}_{count}\;=\;\sum_{j=0}^{r_{\text{max}}}\frac{1}{\bar{r}}\frac{N^{\left(j\right)}}{N}\sum_{i=1}^{m}{\hat{q}}_{i}^{\left(j\right)}\log\frac{{\hat{q}}_{i}^{\left(j\right)}}{{\hat{p}}_{i}}\;. \end{array}$$ (36)

Comparison with the LNC model log-likelihood (Equation 30) reveals that:

$$\begin{array}{c} {\hat{I}}_{count}=\frac{1}{n_{sp}}{ℒ}_{lnc}+\frac{1}{\bar{r}}\hat{H}\left[r\right] \end{array}$$ (37)

where $\hat{H}[r]=-\sum_{j=0}^{r_{\text{max}}}\frac{N^{\left(j\right)}}{N}\log\frac{N^{\left(j\right)}}{N}$ is the plug-in estimate for the marginal entropy of the observed spike counts. Note that this reduces to the relationship between Bernoulli information and LNB model log-likelihood (Equation 20) in the special case where *r*_max_ = 1.

Thus, even in the most general case of an arbitrary distribution over spike counts given a stimulus, the subspace projection *K* that maximizes the histogram-based estimate of mutual information is identical to the maximum-likelihood *K* for an LN model with a corresponding piece-wise constant parametrization of the nonlinearities.

#### Failures of MID under non-Poisson count distributions

We formulate two simple examples to illustrate the sub-optimality of standard MID for neurons whose stimulus-conditioned count distributions are not Poisson. For both examples, the neuron was sensitive to a one-dimensional projection along the horizontal axis and emitted either 0, 1, or 2 spikes in response to a stimulus.

**Fig 7:**
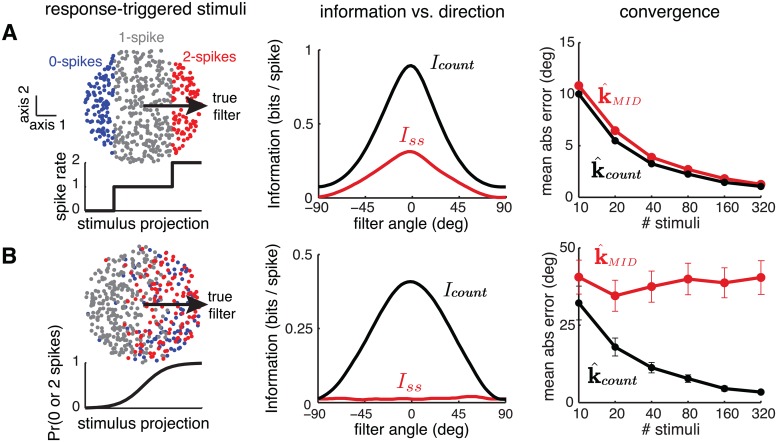
Two examples illustrating sub-optimality of MID under discrete (non-Poisson) spiking. In both cases, stimuli were uniformly distributed within the unit circle and the simulated neuron’s response depended on a 1D projection of the stimulus onto the horizontal axis (*θ* = 0). Each stimulus evoked 0, 1, or 2 spikes. **(A)** Deterministic neuron. *Left*: Scatter plot of stimuli labelled by number of spikes evoked, and the piece-wise constant nonlinearity governing the response (below). The nonlinearity sets the response count deterministically, thus dramatically violating Poisson expectations. *Middle*: information vs. axis of projection. The total information *I*_*count*_ reflects the information from 0-, 1-, and 2-spike responses (treated as distinct symbols), while the single-spike information *I*_*ss*_ ignores silences and treats 2-spike responses as two samples from *p*(**s**|*spike*). *Right*: Average absolute error in ${\hat{\text{k}}}_{MID}$ and ${\hat{\text{k}}}_{count}$ as a function of sample size; the latter achieves 18% lower error due to its sensitivity to the non-Poisson structure of the response. **(B)** Stochastic neuron with sigmoidal nonlinearity controlling the stochasticity of responses. The neuron transitions from almost always emitting 1 spike for large negative stimulus projections, to generating either 0 or 2 spikes with equal probability at large positive projections. Here, the nonlinearity does not modulate the mean spike rate, so *Î*_*ss*_ is approximately zero for all stimulus projections (middle) and the MID estimator does not converge (right). However, the ${\hat{\text{k}}}_{count}$ estimate converges because the LNC model is sensitive to the change in conditional response distribution. Equation (37) details the relationship between *I*_*count*_ and ℒ_*lnc*_, so that this figure can be interpreted from either an information-theoretic or likelihood-based perspective.

Both are illustrated in Fig. 7. The first example (A) involves a deterministic neuron, where spike count is 0, 1, or 2 according to a piece-wise constant nonlinear function of the projected stimulus. Here, MID does not use the information from zero or two-spike bins optimally; it ignores information from zero-spike responses entirely, and treats stimuli eliciting two spikes as two independent samples from *p*(**x**|*spike*). The *I*_*count*_ estimator, by contrast, is sensitive to the non-Poisson statistics of the response, and combines information from all spike counts (Equation 35), yielding both higher information and faster convergence to the true filter.

Our second example (Fig. 7B) involves a model neuron in which a sigmoidal nonlinearity determines the probability that it fires exactly 1 spike (high at negative stimulus projections) or stochastically emits either 0 or 2 spikes, each with probability 0.5 at positive stimulus projections). Thus, the nonlinearity does not change the mean spike rate, but strongly affects its variance. Because the probability of observing a single spike is not affected by the stimulus, single-spike information is zero for all projections, and the MID estimate does not converge to the true filter even with infinite data. However, the full count information *Î*_*count*_ correctly weights the information carried by different spike counts and provides a consistent estimator for *K*.

### Identifying high-dimensional subspaces

A significant drawback to standard MID is that it does not scale tractably to high-dimensional subspaces; that is, to the simultaneous estimation of many filters. MID has usually been limited to estimation of only one or two filters, and we are unaware of a practical setting in which it has been used to recover more than three. This stands in contrast to methods like spike-triggered covariance (STC) [1, 7], information-theoretic spike-triggered average and covariance (iSTAC) [19], projection-pursuit regression [28], Bayesian spike-triggered covariance [14], and quadratic variants of MID [21, 22], all of which can tractably estimate ten or more filters. This capability may be important, given that V1 neurons exhibit sensitivity to as many as 15 dimensions [29], and many canonical neural computations (e.g., motion estimation) require a large number of stimulus dimensions [22, 30].

Before we continue, it is helpful to consider *why* MID is impractical for high-dimensional feature spaces. The problem isn’t the number of filter parameters: these scale linearly with dimensionality, since a *p*-filter model with *D*-dimensional stimuli requires only *Dp* parameters, or indeed only $\left(D-1)p-\frac{1}{2}p(p-1\right)$ parameters to specify the subspace naively after accounting for degeneracies. The problem is instead the number of parameters needed to specify the densities *p*(**x**) and *p*(**x**|*spike*). For histogram-based density estimators, the number of parameters grows exponentially with dimension: a histogram with *m* bins along each of *p* filter axes requires *m* ^*p*^ parameters, a phenomenon sometimes called the “curse of dimensionality”.

#### Density vs. nonlinearity estimation

A key benefit of the LNP model likelihood framework is that it shifts the focus of estimation away from the separate densities *p*(**x**|*spike*) and *p*(**x**) to a single nonlinear function *f*. This change in focus makes it easier to scale the likelihood approach to high dimensions for a few different reasons. First, direct estimation of a single nonlinearity in place of two densities immediately halves the number of parameters required to achieve a similarly detailed picture of the neuron’s response to the filtered stimulus. Second, the dependence of the MID cost function on the logarithm of the ratio *p*(**x**|*spike*)/*p*(**x**) makes it very sensitive to noise in the estimated value of the denominator *p*(**x**) when that value is near 0. Unfortunately, as *p*(**x**) is also the probability with which samples are generated, these low-value regions are precisely where the fewest samples are available. This is a common difficulty in the empirical estimation of information-theoretic quantities, and others working in more general machine-learning settings have suggested direct estimation of the ratio rather than its parts [31–33]. In LN neural modeling such direct estimation of the ratio is equivalent to direct estimation of the nonlinearity.

Third, … as the nonlinearity is a property of the neuron rather than the stimulus, it may be more straightforward to construct a smoothed or structured parametrization for *f* (or to regularize its estimate based on prior beliefs about neuronal properties) instead of regularizing the estimates of stimulus densities. For example, consider an experiment using natural visual images. While natural images presumably form a smooth manifold within the space of all possible pixel patterns, the structure of this manifold is neither simple nor known. The natural distribution of images does not factor over disjoint sets of pixels, nor over linear projections of pixel values. A small random perturbation in all pixels makes a natural image appear unnaturally noisy, violating the underlying presumption of kernel density estimators that local perturbations do not alter the density much. Indeed the question of how best to model the distribution of natural stimuli is a matter of active research. By contrast, we might expect to be able to develop better parametric forms to describe the non-linearities employed by neural systems. For instance, we might expect the neural nonlinearity to vary smoothly in the space of photoreceptor activation, and thus of filter outputs. Thus, locally kernel-smoothed estimates of the non-linear mapping—or even parametric choices of function class, such as low-order polynomials—might be valid, even if the stimulus density changes abruptly. Alternatively, subunits within the neural receptive field might lead to additively or multiplicatively separable components of the nonlinearity that act on the outputs of different filters. In this case, it would be possible to factor *f* between two subsets of filter outputs, say to give *f*(**x**) = *f*_1_(**x**_1_)*f*_2_(**x**_2_), even though there is no reason for the stimulus distribution to factor *p*(**x**) ≠ *p*(**x**_1_)*p*(**x**_2_). This reduction of *f* to two (or more) lower-dimensional functions would avoid the exponential parameter explosion implied by the curse of dimensionality.

Indeed, such strategies for parametrization of the nonlinear mapping are already implicit in likelihood-based estimators inspired by the spike-triggered average and covariance. In many such cases, *f* is parametrized by a quadratic form embedded in a 1D nonlinearity [14], so that the number of parameters scales only quadratically with the number of filters. A similar approach has been formulated in information-theoretic terms using a quadratic logistic Bernoulli model [21, 22, 24]. Another method, known as extended projection pursuit regression (ePPR) [28], parametrizes *f* as a sum of one-dimensional nonlinearities, in which case the number of parameters grows only linearly with the number of filters.

#### Parametrizing the many-filter LNP model

Here we provide a general formulation that encompasses both standard MID and constrained methods that scale to high-dimensional subspaces. We can rewrite the LNP model (Equation 1) as follows:

$$\begin{array}{c} x=K^{⊤}s\;\left(\text{dimensionality reduction}\right) \end{array}$$ (38)

$$\begin{array}{c} \lambda =f\left(x\right)=g\left(\sum_{i=1}^{n_{\phi }}\alpha_{i}\phi_{i}\left(x\right)\right)\;\left(\mathrm{nonlinearity}\right) \end{array}$$ (39)

$$\begin{array}{c} r|\lambda ∼Poiss\left(\lambda \Delta \right)\left(spiking\right). \end{array}$$ (40)

The nonlinearity *f* is parametrized using basis functions {*φ*_*i*_(⋅)}, *i* = 1,…,*n*_*φ*_, which are linearly combined with weights *α*_*i*_ and then passed through a scalar nonlinearity *g*. We refer to *g* as the output nonlinearity; its primary role is to ensure the spike rate *λ* is positive regardless of weights *α*_*i*_. This can also be considered a special case of an LNLN model [15, 34, 35].

If we fix *g* and the basis functions {*φ*_*i*_} in advance, fitting the nonlinearity simply involves estimating the parameters *α*_*i*_ from the projected stimuli and associated spike counts. If *g* is convex and log-concave, then the log-likelihood is concave in {*α*_*i*_} given *K*, meaning the parameters governing *f* can be fit without getting stuck in non-global maxima [11].

Standard MID can be seen as a special case of this general framework: it sets *g* to the identity function and the basis functions *φ*_*i*_ to histogram-bin indicator functions (denoted **1**_*B*_*i*__(⋅) in Equation 7). The maximum-likelihood weights $\left\{{\hat{\alpha }}_{i}\right\}$ are proportional to the ratio between the number of spikes and number of stimuli in the *i*’th histogram bin (Equation 10). As discussed above, the number of basis functions *n*_*φ*_ scales exponentially with the number of filters, making this parametrization impractical for high-dimensional feature spaces.

Another special case of this framework corresponds to Bayesian spike-triggered covariance analysis [14], in which the basis functions *φ*_*i*_ are taken to be linear and quadratic functions of the projected stimulus. If the stimulus is Gaussian, then standard STC and iSTAC provide an asymptotically optimal fit to this model under the assumption that *g* is exponential [14, 19].

In principle, we can select any set of basis functions. Other reasonable choices include polynomials, sigmoids, sinusoids (i.e., Fourier components), cubic splines, radial basis functions, or any mixture of these bases. Alternatively, we could use non-parametric models such as Gaussian processes, which have been used to model low-dimensional tuning curves and firing rate maps [36, 37]. Theoretical convergence for arbitrary high-dimensional nonlinearities requires a scheme for increasing the complexity of the basis or non-parametric model as we increase the amount of data recorded [38–41]. We do not examine such theoretical details here, focusing instead on the problem of choosing a particular basis that is well suited to the dataset at hand. Below, we introduce basis functions {*φ*_*i*_} that provide a reasonable tradeoff between flexibility and tractability for parametrizing high-dimensional nonlinear functions.

#### Cylindrical basis functions for the LNP nonlinearity

We propose to parametrize the nonlinearity for many-filter LNP models using cylindrical basis functions (CBFs), which we introduce by analogy to radial basis functions (RBFs). These functions are restricted in some directions of the feature space (like RBFs), but are constant along other dimensions. They are therefore the function-domain analogues to the probability “experts” used in product-of-experts models [42] in that they constrain a high-dimensional function along only a small number of dimensions, while imposing no structure on the others.

We define a “first-order” CBF as a Gaussian bump in one direction of the feature space, parametrized by center location *μ* and a characteristic width *σ*:

$$\begin{array}{c} \phi_{1st}\left(x\right)=\exp\left(\frac{-{\left(x_{i}-\mu \right)}^{2}}{2\sigma^{2}}\right), \end{array}$$ (41)

which affects the function along vector component *x*_*i*_ and is invariant along *x*_*j* ≠ *i*_. Parametrizing *f* with first-order CBFs is tantamount to assuming *f* can be parametrized as the sum of 1D functions along each filter axis, that is *f*(**x**) = *g*(*f*_1_(*x*_1_) + … *f*_*m*_(*x*_*m*_)), where each function *f*_*i*_ is parametrized with a linear combination of “bump” functions. This setup resembles the parametrization used in the extended projection-pursuit regression (ePPR) model [28], although the nonlinear transformation *g* confers some added flexibility. For example, we can have multiplicative combination when *g*(⋅) = exp(⋅), resulting in a separable *f*, or rectified additive combination when *g*(⋅) = max(⋅,0), which is closer to ePPR. If we use *d* basis functions along each filter axis, the resulting nonlinearity requires *kd* parameters for a *k*-filter LNP model.

We can define second-order CBFs as functions that have Gaussian dependence on two dimensions of the input space and that are insensitive to all others:

$$\begin{array}{c} \phi_{2nd}\left(x\right)=\exp\left(\frac{-{\left(x_{i}-\mu_{i}\right)}^{2}-{\left(x_{j}-\mu_{j}\right)}^{2}}{2\sigma^{2}}\right) \end{array}$$ (42)

where *μ*_*i*_ and *μ*_*j*_ determine the center of the basis function in the (*x*_*i*_,*x*_*j*_) plane. A second-order basis represents *f* as a (transformed) sum of these bivariate functions, giving $\left(\begin{array}{c} k\\ 2 \end{array}\right)d^{2}$ parameters if we use *d* ^2^ basis functions for each of the $\left(\begin{array}{c} k\\ 2 \end{array}\right)$ possible pairs of *k* filter outputs, or merely $\frac{k}{2}d^{2}$ if we instead partition the *k* filters into disjoint pairs. Higher-order CBFs can be defined analogously: *k* ^′^th order CBFs are Gaussian RBFs in a *k* ^′^-dimensional subspace while remaining constant in the remaining *k*−*k* ^′^ dimensions. Of course, there is no need to represent the entire nonlinearity using CBFs of the same order. It might make sense, for example, to represent the nonlinear combination of the first two filter responses with second order CBFs (which is comparable to standard MID with a 2D histogram representation of the nonlinearity), and then use first order CBFs to represent the contributions of additional (less-informative) filter outputs.

To illustrate the feasibility of this approach, we applied dimensionality reduction methods to a previously published dataset from macaque V1 [29]. This dataset contains extracellular single unit recordings of simple and complex cells driven by an oriented 1D binary white noise stimulus sequence (i.e., “flickering bars”). For each neuron, we fit an LNP model using: (1) the information-theoretic spike-triggered average and covariance (iSTAC) estimator [19]; and (2) the maximum likelihood estimator for an LNP model with nonlinearity parametrized by first-order CBFs. The iSTAC estimator, which combines information from the STA and STC, returns a list of filters ordered by informativeness about the neural response. It models the nonlinearity as an exponentiated quadratic function (an instance of a generalized quadratic model [23]), and yields asymptotically optimal performance under the condition that stimuli are Gaussian. For comparison, we also implemented a model with a less-constrained nonlinearity, using Gaussian RBFs sensitive to all filter outputs (rbf-LNP). This approach is comparable to “classic” MID, although it exploits the LNP formulation to allow local smoothing of the nonlinearity (rather than square histogram bins.). Even so, the number of parameters in the nonlinearity still grows exponentially with the number of filters so, computational concerns prevented us from recovering more than four filters with this method.

**Fig 8:**
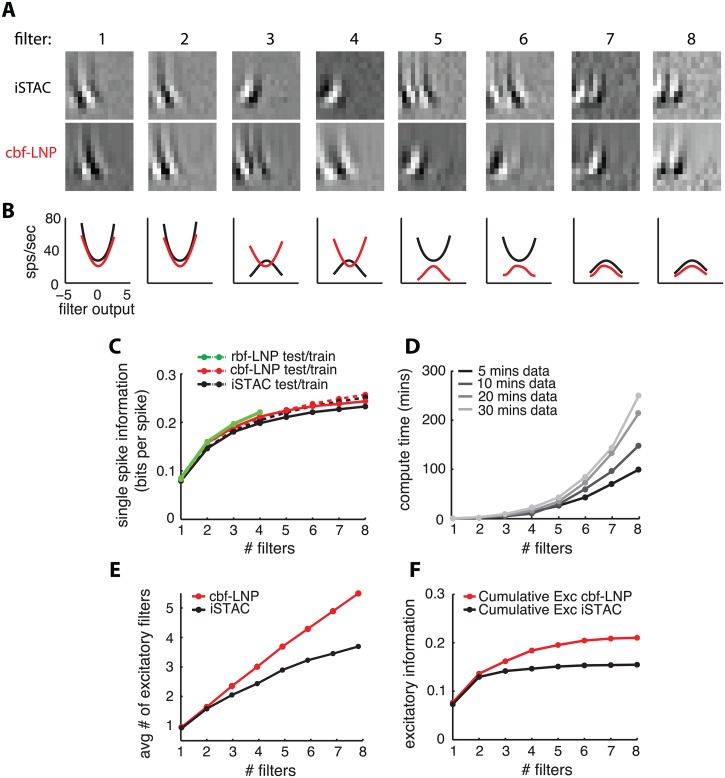
Estimation of high-dimensional subspaces using a nonlinearity parametrized with cylindrical basis functions (CBFs). **(A)** Eight most informative filters for an example complex cell, estimated with iSTAC (*top row*) and cbf-LNP (*bottom row*). For the cbf-LNP model, the nonlinearity was parametrized with three first-order CBFs for the output of each filter (see Methods). **(B)** Estimated 1D nonlinearity along each filter axis, for the filters shown in (A). Note that third and fourth iSTAC filters are suppressive while third and fourth cbf-LNP filter are excitatory. **(C)** Cross-validated single-spike information for iSTAC, cbf-LNP, and rbf-LNP, as a function of the number of filters, averaged over a population of 16 neurons (selected from [29] for having ≥ 8 informative filters). The cbf-LNP estimate outperformed iSTAC in all cases, while rbf-LNP yielded a slight further increase for the first four dimensions. **(D)** Computation time for the numerical optimization of the cbf-LNP likelihood for up to 8 filters. Even for 30 minutes of data and 8 filters, optimisation took about 4 hours. **(E)** Average number of excitatory filters as a function of total number of filters, for each method. **(F)** Information gain from excitatory filters, for each method, averaged across neurons. Each point represents the average amount of information gained from adding an excitatory filter, as a function of the number of filters.

Fig. 8 compares the performance of these estimators on neural data, and illustrates the ability to tractably recover high-dimensional feature spaces using maximum likelihood methods, provided that the nonlinearity is parametrized appropriately. We used 3 CBFs per filter output for the cbf-LNP model (resulting in 3*p* parameters for the nonlinearity), and a grid with 3 RBFs per dimension for the rbf-LNP model (3^*p*^ parameters). By contrast, the exponentiated-quadratic nonlinearity underlying the iSTAC estimator requires *O*(*p* ^2^) parameters.

To compare performance, we analyzed the growth in empirical single-spike information (computed on a “test” dataset) as a function of the number of filters. Note that this is equivalent to computing test log-likelihood under the LNP model. For a subset of neurons determined to have 8 or more informative filters (16/59 cells), the cbf-LNP filters captured more information than the iSTAC filters (Fig. 8C). This indicates that the CBF nonlinearity captures the nonlinear mapping from filter outputs to spike rate more accurately than an exponentiated quadratic, and that this flexibility confers advantages in identifying the most informative stimulus dimensions. The first four filters estimated under the rbf-LNP model captured slightly more information again than the cbf-LNP filters, indicating that first-order CBFs provide slightly too restrictive a parametrization for these neurons. Due to computational considerations, we did not attempt to fit the rbf-LNP model with > 4 filters, but note that the cbf-LNP model scaled easily to 8 filters (Fig. 8D).

In addition to its quantitative performance, the cbf-LNP estimate exhibited a qualitative difference from iSTAC with regard to the ordering of filters by informativeness. In particular, the cbf-LNP fit reveals that excitatory filters provide more information than iSTAC attributes to them, and that excitatory filters should come earlier relative to suppressive filters when ordering by informativeness. Fig. 8A-B, which shows the first 8 filters and associated marginal one-dimensional nonlinearities for an example V1 complex cell, provides an illustration of this discrepancy. Under the iSTAC estimate (Fig. 8A, top row), the first two most informative filters are excitatory but the third and fourth are suppressive (see nonlinearities in Fig. 8B). However, the cbf-LNP estimate (and rbf-LNP estimate, not shown) indicates that the four most informative filters are all excitatory. This tendency holds across the population of neurons. We can quantify it in terms of the number of excitatory filters within the first *n* filters identified (Fig. 8E) or the total amount of information (i.e., log-likelihood) contributed by excitatory filters (Fig. 8F). This shows that iSTAC, which nevertheless provides a computationally inexpensive initialization for the cbf-LNP estimate, does not accurately quantify the information contributed by excitatory filters. Most likely, this reflects the fact that an exponentiated-quadratic does not provide as accurate a description of the nonlinearity along excitatory stimulus dimensions as can be obtained with a non-parametric estimator.

### Relationship to previous work

A variety of neural dimensionality reduction methods have been proposed previously. Here, we consider the relationship of the methods described in this study to these earlier approaches. Rapela *et al* [28] introduced a technique known as extended Projection Pursuit Regression (ePPR), where the high-dimensional estimation problem is reduced to a sequence of simpler low-dimensional ones. The approach is iterative. A one-dimensional model is found first, and the dimensionality is then progressively increased to optimize a cost function, but with the search for filters restricted to dimensions orthogonal to all the filters already identified. From a theoretical perspective this assumes that the spiking probability can be defined as a sum of functions of the different stimulus components; that is,

$$\begin{array}{c} p\left(spike|s\right)=g_{1}\left(k_{1}^{⊤}s\right)+g_{2}\left(k_{2}^{⊤}s\right)+\cdots g_{N}\left(k_{N}^{⊤}s\right)\;. \end{array}$$ (43)

Rowekamp *et al* [43] compared such an approach to the joint optimization more common in MID analysis (as in [18]), and derived the bias that results from sequential optimization and its implicit additivity. By contrast, we have focused here on parametrization rather than sequential optimization. In all cases, we optimized the log-likelihood simultaneously over all filter dimensions. For high-dimensional models, we advocate parametrization of the nonlinearity so as to avoid the curse of dimensionality. However, the CBF form we have introduced is more flexible than that of ePPR, both in that two- or more dimensional components are easily included, and in that the outputs of the components can be combined non-linearly.

Other proposals can be seen as assuming specific quadratic-based parametrizations for the nonlinearity, that are more restrictive than the CBF form. The iSTAC estimator, introduced by Pillow & Simoncelli [19], is based on maximization of the KL divergence between Gaussian approximations to the spike-triggered and stimulus ensembles—thus finding the feature space that maximizes the single-spike information under a Gaussian model of both the spike-triggered and stimulus ensembles. Park & Pillow [44] showed its relationship to an LNP model with an exponentiated quadratic spike rate, which takes the form:

$$\begin{array}{c} p\left(spike|s\right)=\exp\left(a+K^{⊤}s+s^{⊤}Cs\right). \end{array}$$ (44)

Such a nonlinearity readily yields maximum likelihood estimators based on the STA and STC. Moreover, they proposed a new model, known as “elliptical LNP”, which allowed estimation of a non-parametric nonlinearity around the quadratic function (instead of assuming an exponential form). Rajan et al. [24] considered a similar model within an information-theoretic framework and proposed extending it to nonlinear combinations of outputs from multiple quadratic functions. In a similar vein, Sharpee *et al*[45, 46] used

$$\begin{array}{c} p\left(spike|s\right)=\frac{1}{1+\exp(a+Ks+s^{⊤}Cs)}. \end{array}$$ (45)

This model corresponds to quadratic logistic regression, and thus assumes Bernoulli output noise (and a binary response). The authors also proposed a “nonlinear MID” in which the standard MID estimator is extended to by setting the firing rate to be a quadratic function of the form *f*(**k** ^⊤^ **s**+**s** ^⊤^ *C* **s**). This method is one-dimensional in a quadratic stimulus space (unlike multidimensional linear MID) and therefore avoids the curse of dimensionality. Other work has used independent component analysis to find directions in stimulus space in which the spike-triggered distribution has maximal deviations from Gaussianity [8].

## Discussion

### Distributional assumptions implicit in MID

We have studied the estimator known as maximally informative dimensions (MID), [18] a popular approach for estimating informative dimensions of stimulus space from spike-train data. Although the MID estimator was originally described in information-theoretic language, we have shown that, when used with plug-in estimators for information-theoretic quantities, it is mathematically identical to the maximum likelihood estimator for a linear-nonlinear-Poisson (LNP) encoding model. This equivalence holds irrespective of spike rate, the amount of data, or the size of time bins used to count spikes. We have shown that this follows from the fact that the plug-in estimate for single-spike information is equal (up to an additive constant) to the log-likelihood per spike of the data under an LNP model.

Estimators defined by the optima of information-theoretic functionals have attractive theoretical properties, including that they provide well-defined and (theoretically) distribution-agnostic characterizations of data. In practice, however, such agnosticism can be difficult to achieve, as the need to estimate information-theoretic quantities from data requires the choice of a particular estimator. MID has the virtue of using a non-parametric estimator for raw and spike-triggered stimulus densities, meaning that the number of parameters (i.e., the number of histogram bins) can grow flexibly with the amount of data. This allows it to converge for arbitrary densities, in the limit of infinite data. However, for a finite dataset, the choice of number of bins is critical for obtaining an accurate estimate. As we show in Fig. 3, a poor choice can lead to a systematic under- or over-estimate of the single-spike information, and in turn, a poor estimate of the most informative stimulus dimensions. Determining the number of histogram bins should therefore be considered a model selection problem, validated with a statistical procedure such as cross-validation.

A second kind of distributional assumption arises from MID’s reliance on single-spike information, which is tantamount to an assumption of Poisson spiking. To be clear, the single-spike information represents a valid information-theoretic quantity that does not explicitly assume any model. As noted in [26], it is simply the information carried by a single spike time, independent of all other spike times. However, conditionally independent spiking is also the fundamental assumption underlying the Poisson model and, as we have shown, the standard MID estimator (based on the KL-divergence between histograms) is mathematically identical to the maximum likelihood estimator for an LNP model with piece-wise constant nonlinearity. Thus, MID achieves no more and no less than a maximum likelihood estimator for a Poisson response model. As we illustrate in Fig. 4, MID does not maximize the mutual information between the projected stimulus and the spike response when the distribution of spikes conditioned on stimuli is not Poisson; it is an inconsistent estimator for the relevant stimulus subspace in such cases.

The distributional-dependence of MID should therefore be considered when interpreting its estimates of filters and nonlinearities. MID makes different, but not necessarily fewer, assumptions when compared to other LN estimators. For instance, although the maximum-likelihood estimator for a generalized linear model assumes a less-flexible model for the neural nonlinearity than does MID, it readily permits estimation of certain forms of spike-interdependence that MID neglects. In particular, MID-derived estimates are subject to concerns regarding model mismatch that arise whenever the true generative family is unknown [47].

In light of the danger that these distributional assumptions be obscured by the information-theoretic framing of MID, our belief is that the safer approach is to specify a model explicitly and adopt a likelihood-based estimation framework. Where the information theoretic and likelihood-based estimators are identical, nothing is lost by this approach. However, besides making assumptions explicit, the likelihood-based framework also readily facilitates the introduction of suitable priors for regularization of suitable priors, or hierarchical models [48, 49], or more structured models of the type discussed here.

### Generalizations

Having clarified the relationship between MID and LNP model the, we introduced two generalizations designed to recover a maximally informative stimulus projection when neural response variability is not well described as Poisson. From a model-based perspective, the generalizations correspond to maximum likelihood estimators for a linear-nonlinear-Bernoulli (LNB) model (for binary spike counts), and the linear-nonlinear-Count (LNC) model (for arbitrary discrete spike counts). For both models, we obtained an equivalent relationship between log-likelihood and an estimate of mutual information between stimulus and response. This correspondence extends previous work that showed only approximate or asymptotic relationships between between information-theoretic and maximum-likelihood estimators [20, 24, 25]. The LNC model is the most general of the models we have considered. It requires the fewest assumptions, since it allows for arbitrary distributions over spike count given the stimulus. It includes both LNB and LNP as special cases (i.e., when the count distribution is Bernoulli or Poisson, respectively).

We could analogously define arbitrary “LNX” models, where *X* stands in for any probability distribution over the neural response (analog or discrete), and perform dimensionality reduction by maximizing likelihood for the filter parameters under this model. The log-likelihood under any such model can be associated with an information-theoretic quantity, analogous to single-spike, Bernoulli, and count information, using the difference of log-likelihoods (see also [35]):

$$\begin{array}{c} I_{lnx}\;≜\;\sum_{r,s}p\left(s\right)p_{x}\left(r|s,\theta \right)\log p_{x}\left(r|s,\theta \right)\;-\;\sum_{r}p_{x}\left(r|\theta_{0}\right)\log p_{x}\left(r|\theta_{0}\right), \end{array}$$ (46)

where *p*_*x*_(*r*|**s**,*θ*) denotes the conditional response distribution associated with the LNX model with parameters *θ*, and *p*_*x*_(*r*|*θ*_0_) describes the marginal distribution over *r* under the stimulus distribution *p*(**s**). The empirical or plug-in estimate of this information is equal to the LNX model log-likelihood plus the estimated marginal entropy:

$$\begin{array}{c} {\hat{I}}_{lnx}\left(\theta \right)=\frac{1}{n}\left({ℒ}_{lnx}\left(\theta ;D\right)-{ℒ}_{lnx}\left(\theta_{0};D\right)\right), \end{array}$$ (47)

where *n* denotes the number of samples and *θ*_0_ depends only on the marginal response distribution. The maximum likelihood estimate is therefore equally a maximal-information estimate.

Note that all of the dimensionality-reduction methods we have discussed treat neural responses as conditionally independent given the stimulus, meaning that they do not capture dependencies between spike counts in different time bins (e.g., due to refractoriness, bursting, adaptation, etc.). Spike-history dependencies can influence the single-bin spike count distribution—for example, a Bernoulli model is more accurate than a Poisson model when the bin size is smaller than or equal to the refractory period, since the Poisson model assigns positive probability to the event of having ≥ 2 two spikes in a single bin. The models we have considered can all be extended to capture spike history dependencies by augmenting the stimulus with a vector representation of spike history, as in both conditional renewal models and generalized linear models [10, 12, 27, 50–52].

Lastly, we have shown that viewing MID from a model-based perspective provides insight into how to overcome practical limitations on the number of filters that can be estimated. Standard implementations of MID employ histogram-based density estimators for *p*(*K* ^⊤^ **s**) and *p*(*K* ^⊤^ **s**|*spike*). However, dimensionality and parameter count can be a crippling issue given limited data, and density estimation becomes intractable in dimensionalities > 3. Furthermore, the dependence of the information on the logarithm of the ratio of these densities amplifies sensitivity to errors in these estimates. The LNP-likelihood view suggests direct estimation of the nonlinearity *f*, rather than of the densities. Such estimates are naturally more robust, and are more sensibly regularized based on expectations about neuronal responses without reference to any regularities in the stimulus distribution. We have proposed a flexible yet tractable form for the nonlinearity in terms of linear combinations of basis functions cascaded with a second *output* nonlinearity. This approach yielded a flexible, computationally efficient, constrained version of MID that is able to estimate high-dimensional feature spaces. It is also general in the sense that it encompasses standard MID, generalized linear and quadratic models, and other constrained models that scale tractably to high-dimensional subspaces. Future work might seek to extend this flexible likelihood-based approach further, for example by including priors over the weights with which basis functions are combined to improve regularization, or perhaps by adjusting hyperparameters in a hierarchical model as has been successful with linear approaches [48, 49].

In recent years, the ability to successfully characterize low-dimensional neural feature spaces using MID has proved useful to address questions relating to multidimensional feature selectivity [53–56]. In all of these examples however, issues with dimensionality have prevented the estimation of feature spaces with more than two dimensions. The methods presented within this paper will help to overcome these issues, opening access to further important questions regarding the relationship between stimuli and their neural representation.

## Methods

### Bound on lost information under MID

Here we present a derivation of the lower bound on the fraction of total information carried by silences for a Bernoulli neuron, in the limit of rare spiking. For notational convenience, let *ρ* = *p*(*r* = 1) denote the marginal probability of a spike, so the probability of silence is *p*(*r* = 0) = 1−*ρ*. Let *Q*_1_ = *p*(**s**|*r* = 1) and *Q*_0_ = *p*(**s**|*r* = 0) denote the spike-triggered and silence-triggered stimulus distributions, respectively. Let *P*_**s**_ = *p*(**s**) denote the raw stimulus distribution. Note that we have the *P*_**s**_ = *ρQ*_1_+(1−*ρ*)*Q*_0_. The mutual information between the stimulus and one bin of the response (Equation 18) can then be written

$$\begin{array}{c} I\left(s,r\right)=\rho D_{KL\;}\left(Q_{1}\;|\;|\;P_{s}\right)+\left(1-\rho \right)D_{KL\;}\left(Q_{0}\;|\;|\;P_{s}\right). \end{array}$$ (48)

Note that this is a generalized form of the Jensen-Shannon (JS) divergence; the standard JS-divergence between *Q*_0_ and *Q*_1_ is obtained when $\rho =\frac{1}{2}$.

In the limit of small *ρ* (i.e., the Poisson limit), the mutual information is dominated by the first (*Q*_1_) term. Here we wish to show a bound on the fraction of information carried by the *Q*_0_ term. We can do this by computing a second-order Taylor expansion of $(1-\rho )D_{KL}\left(Q_{o}|P_{s}\right)$ and *I*(**s**,*r*) around *ρ* = 0, and show that their ratio is bounded below by *ρ*/2. Expanding in *ρ*, we have

$$\begin{array}{ccc} \left(1-\rho \right)D_{KL\;}\left(Q_{o}\;|\;|\;P_{s}\right) & = & \frac{1}{2}\rho^{2}V\left(Q_{1},Q_{0}\right)+O\left(\rho^{3}\right),\mathrm{and} \end{array}$$ (49)

$$\begin{array}{ccc} I(s,r) & = & \rho D_{KL\;}\left(Q_{1}\;|\;|\;Q_{0}\right)-\frac{1}{2}\rho^{2}V\left(Q_{1},Q_{0}\right)+O\left(\rho^{3}\right), \end{array}$$ (50)

where

$$\begin{array}{c} V\left(Q_{1},Q_{0}\right)=\int_{\Omega }Q_{1}\left(\frac{Q_{1}}{Q_{0}}-1\right)ds, \end{array}$$ (51)

which is a an upper bound on the KL-divergence: $V(Q_{1},Q_{0})\geq D_{KL\;}(Q_{1}\;|\;|\;Q_{0})$, since (*z*−1) ≥ log(*z*). We therefore have

$$\begin{array}{c} \frac{\left(1-\rho \right)D_{KL\;}\left(Q_{o}\;|\;|\;P_{s}\right)}{I(s,r)}=\frac{\frac{1}{2}\rho^{2}V\left(Q_{1},Q_{0}\right)+O\left(\rho^{3}\right)}{\rho D_{KL\;}\left(Q_{1}\;|\;|\;Q_{0}\right)-\frac{1}{2}\rho^{2}V\left(Q_{1},Q_{0}\right)+O\left(\rho^{3}\right)}\geq \frac{\rho V(Q_{1},Q_{0})}{2D_{KL\;}\left(Q_{1}\;|\;|\;Q_{0}\right)}\geq \frac{\rho }{2} \end{array}$$ (52)

in the limit *ρ* → 0.

We conjecture that the bound holds for all values of *ρ*. For the case of $\rho =\frac{1}{2}$, this corresponds to an assertion about the relative contribution of each of the two terms in the JS divergence, that is:

$$\begin{array}{c} \frac{D_{KL\;}\left(Q_{1}\;|\;|\;\frac{1}{2}\left(Q_{0}+Q_{1}\right)\right)}{D_{KL\;}\left(Q_{1}\;|\;|\;\frac{1}{2}\left(Q_{0}+Q_{1}\right)\right)+D_{KL\;}\left(Q_{1}\;|\;|\;\frac{1}{2}\left(Q_{0}+Q_{1}\right)\right)}\geq \frac{1}{4} \end{array}$$ (53)

for any choice of distributions *Q*_0_ and *Q*_1_. We have been unable to find any counter-examples to this (or to the more general conjecture), but have so far been unable to find a general proof.

### Single-spike information and Poisson log-likelihood

An important general corollary to the equivalence between MID and an LNP maximum likelihood estimate is that the standard single-spike information estimate *Î*_*ss*_ based on a PSTH measured in response to repeated stimuli is also a Poisson log-likelihood per spike (plus a constant). Specifically, the empirical single-spike information is equal to the log-likelihood ratio between an inhomogeneous and homogeneous Poisson model of the repeat data (normalized by spike count):

$$\begin{array}{c} {\hat{I}}_{ss}=\frac{1}{n_{sp}}\left(ℒ\left({\hat{\lambda }}_{ML};r\right)-ℒ\left(\bar{\lambda };r\right)\right), \end{array}$$ (54)

where ${\hat{\lambda }}_{ML}$ denotes the maximum-likelihood or plug-in estimate of the time-varying spike rate (i.e., the PSTH itself), $\hat{\lambda }$ is the mean spike rate across time, and ℒ(**λ**;**r**) denotes the log-likelihood of the repeat data **r** under a Poisson model with time-varying rate **λ**.

We can derive this equivalence as follows. Let {*r*_*jt*_} denote spike counts collected during a “frozen noise” experiment, with repeat index *j* ∈ {1,…,*n*_*rpt*_} and index *t* ∈ {1,…,*n*_*t*_} over time bins of width Δ. Then *T* = *n*_*t*_Δ is the duration of the stimulus and *N* = *n*_*t*_ *n*_*rpt*_ is the total number of time bins in the entire experiment. The single-spike information can be estimated with a discrete version of the formula for single-spike information provided in [26] (see eq. 2.5):

$$\begin{array}{c} {\hat{I}}_{ss}=\frac{1}{n_{t}}\sum_{t=1}^{n_{t}}\frac{\hat{\lambda }\left(t\right)}{\bar{\lambda }}\log\frac{\hat{\lambda }\left(t\right)}{\bar{\lambda }}, \end{array}$$ (55)

where $\hat{\lambda }(t)=\frac{1}{\Delta n_{rpt}}\sum_{j=1}^{n_{rpt}}r_{jt}$ is an estimate of the spike rate in the *t*’th time bin in response to the stimulus sequence *s*, and $\bar{\lambda }=(\sum_{t=1}^{n_{t}}\hat{\lambda }(t))/n_{t}$ is the mean spike rate across the experiment. Note that this formulation assumes (as in [26]) that *T* is long enough that an average over stimulus sequences is well approximated by the average across time.

The plug-in (ML) estimator for spike rate can be read off from the peri-stimulus time histogram (PSTH). It results from averaging the response across repeats for each time bin:

$$\begin{array}{c} \hat{\lambda }\left(t\right)=\frac{1}{n_{rpt}\Delta }\sum_{j=1}^{n_{rpt}}r_{jt}. \end{array}$$ (56)

Clearly, $\bar{\lambda }=\frac{n_{sp}}{N\Delta }$, where *n*_*sp*_ = ∑_*j*,*t*_ *r*_*jt*_ is the total spike count. This allows us to rewrite single-spike information (Equation 55) as:

$$\begin{array}{c} {\hat{I}}_{ss}=\frac{n_{rpt}\Delta }{n_{sp}}\sum_{t=1}^{n_{t}}\hat{\lambda }\left(t\right)\log\hat{\lambda }\left(t\right)-\log\frac{n_{sp}}{N\Delta }. \end{array}$$ (57)

Now, consider the Poisson log-likelihood ℒ evaluated at the ML estimate $\hat{\lambda }=(\hat{\lambda }(1),\ldots ,\hat{\lambda }(n_{t}))$, i.e., the conditional probability of the response data **r** = {*r*_*jt*_} given rate vector $\hat{\lambda }$. This is given by:

$$\begin{array}{ccc} ℒ(\hat{\lambda };r) & = & \sum_{t=1}^{n_{t}}\sum_{j=1}^{n_{rpt}}\left(r_{jt}\log\left(\hat{\lambda }\left(t\right)\Delta \right)-\hat{\lambda }\left(t\right)\Delta -\log r_{jt}!\right)\\ & = & \sum_{t=1}^{n_{t}}\left(\sum_{j=1}^{n_{rpt}}r_{jt}\right)\log\hat{\lambda }\left(t\right)-n_{sp}+n_{sp}\log\Delta -\sum_{t,j}\log r_{jt}!\\ & = & n_{rpt}\Delta \sum_{t=1}^{n_{t}}\hat{\lambda }\left(t\right)\log\hat{\lambda }\left(t\right)-n_{sp}+n_{sp}\log\Delta -\sum_{t,j}\log r_{jt}!\\ & = & n_{sp}{\hat{I}}_{ss}+n_{sp}\log\frac{n_{sp}}{N}-n_{sp}-\sum_{t,j}\log r_{jt}!\\ & = & n_{sp}{\hat{I}}_{ss}+ℒ\left(\bar{\lambda };r\right), \end{array}$$ (58)

which is identical to relationship between single-spike information and Poisson log-likelihood expressed in Equation 13. Thus, even when estimated from raster data, *I*_*ss*_ is equal to the difference between Poisson log-likelihoods under an inhomogeneous (rate-varying) and a homogeneous (constant rate) Poisson model, divided by spike count (see also [57]). These normalized log-likelihoods can be conceived as entropy estimates, with $-\frac{1}{n_{sp}}ℒ(\bar{\lambda };r)$ providing an estimate for prior entropy, measuring the prior uncertainty about spike times given the mean rate, and $-\frac{1}{n_{sp}}ℒ(\hat{\lambda };r)$ corresponding to posterior entropy, measuring the posterior uncertainty once we know the time-varying spike rate.

A similar quantity has been used to report the cross-validation performance of conditionally Poisson models, including the GLM [13, 58]. To penalize over-fitting, the empirical single-spike information is evaluated using the rate estimate $\hat{\lambda }$ obtained with parameters fit to training data and responses **r** from unseen test data. This results in the “cross-validated” single-spike information:

$$\begin{array}{c} {\hat{I}}_{ss}^{\left[xv\right]}=\frac{1}{{n_{sp}}^{\left[test\right]}}\left(ℒ\left({\hat{\lambda }}^{\left[train\right]};r^{\left[test\right]}\right)-ℒ({\bar{\lambda }}^{\left[test\right]};r^{\left[test\right]}\right). \end{array}$$ (59)

This can be interpreted as the predictive information (in bits-per-spike) that the model captures about test data, above and beyond that captured by a homogeneous Poisson model with correct mean rate. Note that this quantity can be negative in cases of extremely poor model fit, that is, when the model prediction on test data is worse than of the best constant-spike-rate Poisson model. Cross-validated single-spike information provides a useful measure for comparing models with different numbers of parameters (e.g., a 1-filter vs. 2-filter LNP model), since units of “bits” are more interpretable than raw log-likelihood of test data. Generally, ${\hat{I}}_{ss}^{\left[xv\right]}$ can be considered to a lower bound on the model’s true predictive power, due to stochasticity in both training and test data. By contrast, the empirical *I*_*ss*_ evaluated on training data tends to over-estimate information due to over-fitting.

### Computation of model-based information quantities

To gain intuition for the different information measures we have considered (Poisson, Bernoulli, and categorical or “count”), it is useful to consider how they differ for a simple idealized example. Consider a world with two stimuli, ‘*A*’ and ‘*B*’, and two possible discrete stimulus sequences, *s*_1_ = *AB* and *s*_2_ = *BA*, each of which occurs with equal probability, so *p*(*s*_1_) = *p*(*s*_2_) = 0.5. Assume each sequence lasts *T* = 2s, so the natural time bin size for considering the spike response is Δ = 1s. Suppose that stimulus *A* always elicits 3 spikes, while *B* always elicits 1 spike. Thus, when sequence *s*_1_ is presented, we observe 3 spikes in the first time interval and 1 spike in the second interval; when *s*_2_ is presented, we observe 1 spike in the first time interval and 3 spikes in the second.

Single-spike information can be computed exactly from *λ*_1_(*t*) and *λ*_2_(*t*), the spike rate in response to stimulus sequence *s*_1_ and *s*_2_, respectively. For this example, *λ*_1_(*t*), takes the value 3 during (0,1] and 1 during (1,2], while *λ*_2_(*t*) takes values 1 and 3 during the corresponding intervals. The mean spike rate for both stimuli is *λ̄* = 2 sp/s. Plugging these into Equation 54 gives single-spike information of *I*_*ss*_ = 0.19 bits/spike. This result is slightly easier to grasp using an equivalent definition of single-spike information as the mutual information between the stimulus *s* and a single spike time *τ* (see [26]). If one were told that a spike, sampled at random from the four spikes present during every trial, occurred during [0, 1], then the posterior *p*(*s*|*τ* = 1) attaches 3/4 probability to *s* = *s*_1_ and 1/4 to *s* = *s*_2_. The posterior entropy is therefore −0.25 log 0.25−0.75 log 0.75 = 0.81 bits. We obtain the same entropy if the spike occurs in the second interval, so *H*(*s*|*τ*) = 0.81. The prior entropy is *H*(*s*) = 1 bit, so once again we have *I*_*ss*_ = 1−0.81 = 0.19 bits/spike.

The Bernoulli information, by contrast, is undefined, since *r* takes values outside the set {0,1}, and therefore cannot have a Bernoulli distribution. To make Bernoulli information well defined, we would need to either truncate spike counts above 1 (*e.g.*, [59]), or else use smaller bin size so that no bin contains more than one spike. In the latter case, we would need to provide more information about the distribution of spike times within these finer bins. If, for example, the three spikes elicited by *A* are evenly spaced within the interval and we use bins equal to 1/3*s*, then the Bernoulli information will clearly exceed single-spike information, since the time of a no-spike response (*r* = 0, a term neglected by single-spike information) provides perfect information about the stimulus, since it occurs only in response to *B*.

Lastly, the count information is easy to compute from the fact that count *r* carries perfect information about the stimulus, so the mutual information between stimulus (*A* or *B*) and *r* is 1 bit. We defined *I*_*count*_ to be the mutual information normalized by the mean spike count per bin (Equation 35). Thus, *I*_*count*_ = 0.5 bits/spike, which is more than double the single-spike information.

### Gradient and Hessian of LNP log-likelihood

Here we provide formulas useful for fitting the the many-filter LNP model with cylindrical basis function (CBF) nonlinearity. We performed joint optimization of filter parameters *K* and basis function weights {*α*_*i*_} using MATLAB’s fminunc function. We found this approach to converge much more rapidly than alternating coordinate ascent. We used analytically computed gradient and Hessian of the joint-likelihood to speed up performance, which we provide here.

Given a dataset ${\left\{\left(s_{t},r_{t}\right)\right\}}_{t=1}^{n_{t}}$, define **r** = (*r*_1_,…,*r*_*n*_*t*__)^⊤^ and **λ** = (*f*(*K* ^⊤^ **s**_1_),…,*f*(*K* ^⊤^ **s**_*n*_*t*__))^⊤^, where nonlinearity *f* = *g*(∑*α*_*i*_ *φ*_*i*_) depends on basis function **Φ** = {*φ*_*i*_} and weights **α** = {*α*_*i*_} (Equation 39). We can write the log-likelihood for the many-filter LNP model (from Equations 38–40) as:

$$\begin{array}{c} ℒ\left(\theta \right)=r^{⊤}\log\lambda -\left(\Delta \right)1^{⊤}\lambda \end{array}$$ (60)

where *θ* = {*K*,**α**} are the model parameters, Δ is the time bin size, and **1** denotes a vector of ones. The first and second derivatives of the log-likelihood are given by

$$\begin{array}{ccc} \frac{\partial ℒ}{\partial \theta_{i}} & = & {\left(\frac{\partial \lambda }{\partial \theta_{i}}\right)}^{⊤}\;\left(\frac{r}{\lambda }-\Delta 1\right) \end{array}$$ (61)

$$\begin{array}{ccc} \frac{\partial^{2}ℒ}{\partial \theta_{i}\partial \theta_{j}} & = & {\left(\frac{\partial^{2}\lambda }{\partial \theta_{i}\partial \theta_{j}}\right)}^{⊤}\;\left(\frac{r}{\lambda }-\Delta 1\right)+{\left(\frac{\partial \lambda }{\partial \theta_{i}}\frac{\partial \lambda }{\partial \theta_{j}}\right)}^{⊤}\;\left(\frac{r}{\lambda^{2}}\right), \end{array}$$ (62)

where multiplication, division, and exponentiation operations on vector quantities indicate component-wise operations.

Let **k**_1_,…,**k**_*m*_ denote the linear filters, i.e., the *m* columns of *K*. Then the required gradients of **λ** with respect to the model parameters can be written:

$$\begin{array}{ccc} \frac{\partial \lambda }{\partial k_{i}} & = & S^{⊤}\left(\lambda^{'}\circ \Phi^{\left(i\right)}\alpha \right) \end{array}$$ (63)

$$\begin{array}{ccc} \frac{\partial \lambda }{\partial \alpha } & = & \Phi^{⊤}\lambda^{'} \end{array}$$ (64)

where *S* denotes the (*n*_*t*_×*D*) stimulus design matrix, Φ denotes the (*n*_*t*_×*n*_*φ*_) matrix whose (*t*,*j*)’th entry is *φ*_*j*_(*K* ^⊤^ **s**_*t*_), and Φ^(*i*)^ denotes a matrix of the same size, formed by the point-wise derivative of Φ with respect to its *i*’th input component, evaluated at each projected stimulus *K* ^⊤^ **s**_*t*_. Finally, **λ** ^′^ = *g* ^′^(Φ**α**) is a (*n*_*t*_×1) vector composed of the point-wise derivatives of the inverse-link function *g* at its input, and ‘∘’ denotes Hadamard or component-wise vector product.

Lastly, second derivative blocks, which can be plugged into Equation 62 to form the Hessian, are given by

$$\begin{array}{ccc} \frac{\partial^{2}\lambda }{\partial k_{i}\partial k_{j}} & = & S^{⊤}\mathrm{diag}\left(\left[\lambda^{''}\circ \left(\Phi^{\left(i\right)}\alpha \right)\circ \left(\Phi^{\left(j\right)}\alpha \right)\right]+\left[\lambda^{'}\circ \Phi^{\left(i,j\right)}\alpha \right]\right)S \end{array}$$ (65)

$$\begin{array}{ccc} \frac{\partial^{2}\lambda }{\alpha^{2}} & = & \Phi^{⊤}\mathrm{diag}\left(\lambda^{''}\right)\Phi \end{array}$$ (66)

$$\begin{array}{ccc} \frac{\partial^{2}\lambda }{\partial k_{i}\partial \alpha } & = & S^{⊤}\left(\mathrm{diag}\left(\lambda^{''}\circ \left(\Phi^{\left(i\right)}\alpha \right)\right)\Phi +\mathrm{diag}\left(\lambda^{'}\right)\Phi^{\left(i\right)}\right), \end{array}$$ (67)

where **λ** ^′′^ = *g* ^′′^(Φ**α**) and Φ^(*i*,*j*)^ is a matrix of point-wise second-derivatives of Φ with respect to *i*’th and *j*’th inputs, evaluated for each projected stimulus *K* ^⊤^ **s**_*t*_.

### V1 data analysis

To examine performance in recovering high-dimensional subspaces, we analyzed data from macaque V1 cells, driven by 1D binary white noise “flickering bars” stimulus, presented at a frame rate of 100 Hz (data published in [29]). The spatiotemporal stimulus had between 8 and 32 spatial bars and we considered 10 time bins for the temporal integration window. This made for a stimulus space with dimensionality ranging from 80 to 320.

The cbf-LNP model was implemented with a cylindrical basis function (CBF) nonlinearity using three first-order CBFs per filter. For a *k*-filter model, this resulted in 3*k* parameters for the nonlinearity, and (240+3)*k* parameters in total for a stimulus with 24 bars.

The traditional MID estimator (rbf-LNP) was implemented using radial basis functions (RBFs) to represent the nonlinearity. Unlike the histogram-based parametrization discussed in the manuscript (which produces a piece-wise constant nonlinearity), this results in a smooth nonlinearity and, more importantly, a smooth log-likelihood with tractable analytic gradients. We defined a grid of RBFs with three grid points per dimension, so that CBF and RBF models were identical for a 1-filter model. For a *k*-filter model, this resulted in 3^*k*^ parameters for the nonlinearity, and 240*k*+3^*k*^ parameters in total.

For both models, the basis function responses were combined linearly and transformed by a “soft-rectification” function: *g*(⋅) = log(1+exp(⋅)), to ensure positive spike rates. We also evaluated the performance of an exponential function, *g*(⋅) = exp(⋅), which yielded slightly worse performance (reducing single-spike information by ∼ 0.02 bits/spike).

The cbf- and rbf-LNP models were both fit by maximizing the likelihood for the model parameters *θ* = {*K*,**α**}. Both models were fit incrementally, with the *N*+1 dimensional model being initialized with the parameters of the *N* dimensional model, plus one additional filter (initialized with the iSTAC filter that provided the greatest increase in log-likelihood). The joint likelihood in *K* and **α** was ascended using MATLAB’s fminunc optimization function, which exploits analytic gradients and Hessians. The models were fit to 80% of the data, with the remaining 20% used for validation.

In order to calculate information contributed by excitatory filters under the cbf-LNP model (Fig. 8F), we removed each filter from the model and refit the nonlinearity (using the training data) using just the other filters. We quantified the information contributed by each filter as the difference between log-likelihood of the full model and log-likelihood of the reduced model (on test data). We sorted the filters by informativeness and computed the cumulative sum of information loss to obtain the trace shown in (Fig. 8F).

Measurements of computation time (Fig. 8D) were averaged over 100 repetitions using different random seeds. For each cell, four segments of activity were chosen randomly with fixed lengths of 5, 10, 20 and 30 minutes, which contained between about 22000 and 173000 spikes. Even with 30 minutes of data, 8 filters could be identified within about 4 hours on a desktop computer, making the approach tractable even for large numbers of filters.

Code will be provided at http://pillowlab.princeton.edu/code.html.
