# Correction: The Equivalence of Information-Theoretic and Likelihood-Based Methods for Neural Dimensionality Reduction

**DOI:** 10.1371/journal.pcbi.1007139

**Published:** 2019-06-14

**Authors:** The PLOS Computational Biology Staff

## Notice of republication

This article was republished on April 23, 2015, to correct errors in the equations that were introduced during the typesetting process. The publisher apologizes for the errors. Please download this article again to view the correct version.

## Supporting information

S1 File Republished, corrected article. (PDF)
